# Renal Clearable Ru-based Coordination Polymer Nanodots for Photoacoustic Imaging Guided Cancer Therapy

**DOI:** 10.7150/thno.36986

**Published:** 2019-10-21

**Authors:** Rui Zhang, Xing Fan, Zhouqi Meng, Haiping Lin, Qiutong Jin, Fei Gong, Ziliang Dong, Youyong Li, Qian Chen, Zhuang Liu, Liang Cheng

**Affiliations:** Institute of Functional Nano & Soft Materials (FUNSOM), Jiangsu Key Laboratory for Carbon-Based Functional Materials and Devices, Soochow University, Suzhou 215123, China.

**Keywords:** Ru-Phen CPNs, ultra-small size nanodots, photoacoustic imaging, photothermal therapy, renal clearance

## Abstract

**Rationale**: Despite the promises of applying theranostic nanoagents for imaging-guided cancer therapy, the chronic retention of these nanoagents may cause safety concerns that hinder their future clinical applications. The metabolizable nanoagents with rapid renal excretion to avoid long-term toxicity is a possible solution for this issue.

**Method**: Herein, we synthesize ultra-small metal-organic coordination polymer nanodots based on ruthenium ion (Ru^3+^) / phenanthroline (Phen) (Ru-Phen CPNs) with superior near-infrared (NIR) absorption. The size, photothermal conversion, cytotoxicity, photoacoustic imaging, *in vivo* &* in vitro* cancer treatment efficiency and biosafety are tested.

**Results**: The size of the ultra-small Ru-Phen CPNs is 6.5 nm. The photothermal conversion efficiency is measured to be ~ 60.69 %, much higher than that of previously reported photothermal agents. The Ru-Phen CPNs could be employed for photoacoustic (PA, 808 nm) imaging-guided photothermal therapy (PTT, 808 nm, 0.5 W/cm^2^) with great performance. Notably, the intrinsic PA signals (808 nm) of Ru-Phen CPNs are observed in kidneys of treated mice, illustrating efficient renal clearance of those ultra-small CPNs. Moreover, the clearance of CPNs is further confirmed by detecting Ru levels in urine and feces.

**Conclusion**: Our work presents a new type of ultra-small Ru-based CPNs with a record high photothermal conversion efficiency, efficient tumor retention after systemic administration, and rapid renal excretion to avoid long-term toxicity, promising for imaging-guided photothermal therapy.

## Introduction

Photothermal therapy (PTT) and photodynamic therapy (PDT) are two typical classes of phototherapies. Under appropriate light irradiation, photothermal agents could produce hyperthermia while photosensitizers could utilize the light energy to activate oxygen to produce cytotoxic reactive oxygen species 1, so as to kill tumors in a minimally invasive manner 1, 2. Moreover, to improve the therapeutic efficiency and accuracy of phototherapy, imaging may be introduced to optimize the therapeutic plan by providing information about the location and size of the tumor, as well as monitoring the *in vivo* behaviors especially the time-dependent tumor uptake of phototherapeutics 3, 4. Although various types of nanoscale theranostic agents have been widely developed for imaging-guided cancer therapy, many challenges still remain to be overcome. On one hand, it is necessary to design nanoagents with higher photothermal conversion efficiency to minimize the energy of incident light 5, 6. On the other hand, the non-biodegradation behavior of most previously reported nanoagents significantly hinder their clinical translation owing to concerns about their potential long-term toxicity 7-9.

Composed of metal ions and organic ligands, coordination polymer nanoparticles featured with inherent biodegradability, ease of surface functionalization and compositional diversity, have emerged as promising multifunctional theranostic nano-platforms 10-13. In recent years, CPNs with different structures, controllable sizes, and abundant chemical / physical functions have been synthesized and applied in biomedicine 14-20. For instance, CPNs containing paramagnetic ions (*e.g.*, Gd^3+^, Mn^2+^) can be used for magnetic resonance imaging (MRI), and those containing high Z elements (*e.g.*, Au, Bi, Hf, W) can be used for computed tomography (CT) imaging or enhanced radiotherapy 17-20. On the other hand, the organic ligands, including fluorescence molecules and chemotherapeutic molecules can be utilized for fluorescence imaging or chemotherapy, respectively 21-23. In particular, ultra-small CPNs with the size of less than 8 nm would undergo fast renal clearance, which can greatly reduce their retention in the body and potential long-term toxicity 15, 18. Therefore, it would be interesting to develop ultra-small CPNs with ultra-high NIR absorbance for imaging-guided photothermal therapy with high precision and effectiveness.

**Figure 1:**
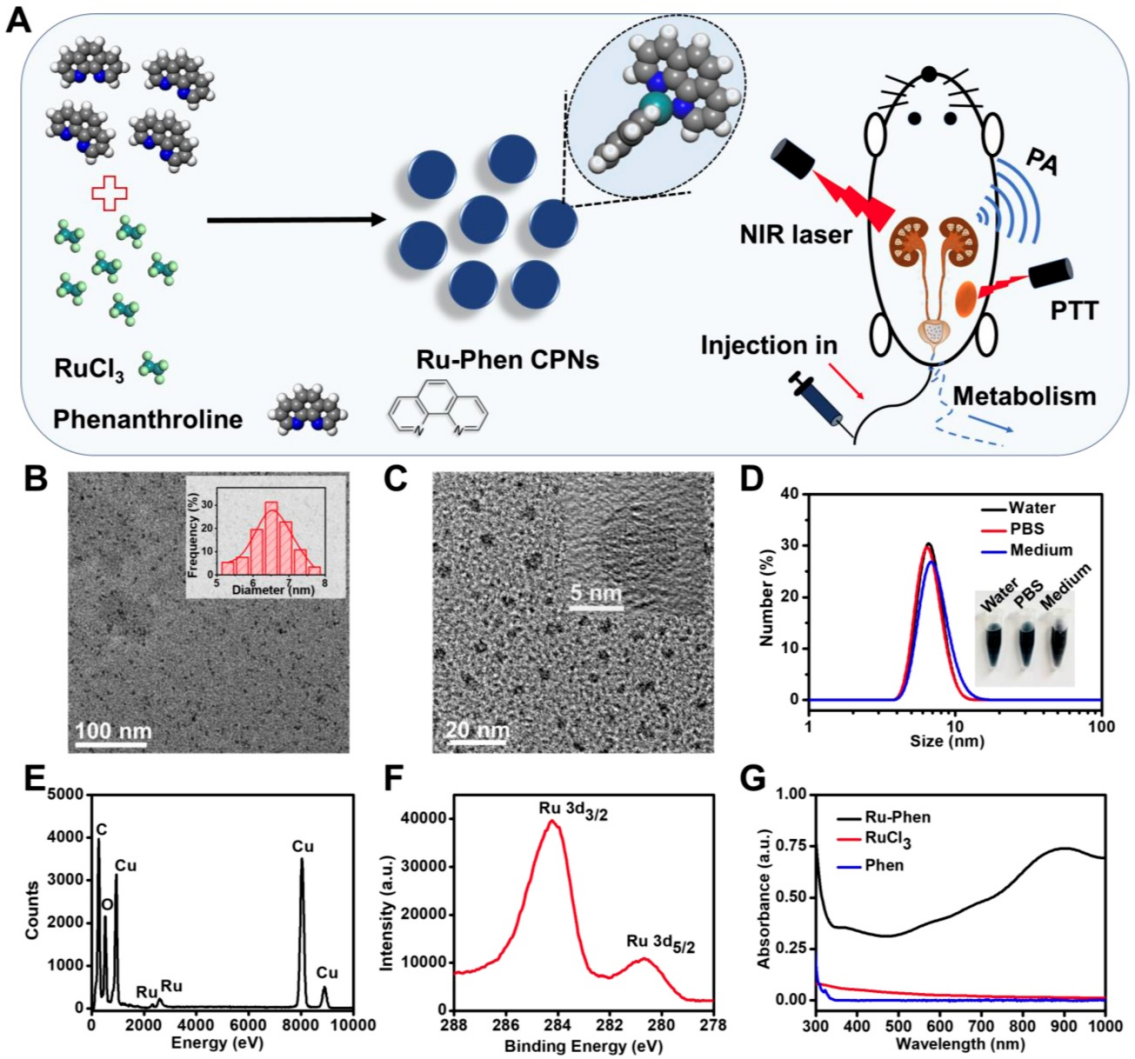
Synthesis and characterization of Ru-Phen CPNs. (**A**) The synthesis pathway, photothermal therapy and renal clearance behavior of Ru-Phen CPNs. (**B&C**) TEM image of Ru-Phen CPNs. Inset in **Figure 1B**: TEM measured diameter distribution of Ru-Phen CPNs. Inset in **Figure 1C**: HRTEM of Ru-Phen CPNs (**D**) DLS data of Ru-Phen CPNs in different solutions. Inset: Optical images of Ru-Phen CPNs in water, PBS, and medium (1640 cell medium + 10 % FBS). (**E**) EDX spectrum of Ru-Phen CPNs. (**F**) XPS spectrum of Ru in Ru-Phen CPNs. (**G**) UV-vis-NIR absorbance spectra of Ru-Phen CPNs, RuCl_3_, and Phen.

Transition-metal-ion-based complexes (cisplatin and other platinum-based anti-cancer drugs) has achieved great progress in the field of biomedicine 24, 25. Beyond the platinum family which has been extensively explored as chemotherapeutics, ruthenium-based complexes have also shown many fascinating features with potential for biomedical applications 26. With the help of different polypyridyl ligands, the formed metal-organic complexes exhibit long-lived excited state after light absorption, promising for applications in photochemistry 27-34. Herein, we fabricate ultra-small Ru-phenanthroline (Ru-Phen) CPNs with a diameter of ~ 6.5 nm, which would satisfy the requirement for renal filtration (**Figure 1A**). Interestingly, despite the insignificant optical absorbance of free Ru^3+^ and Phen, the obtained Ru-Phen CPNs show strong NIR absorbance with an extremely high photothermal conversion efficiency measured to be ~ 60.69 %, which is the highest record among the previously reported photothermal agents. As the results, those Ru-Phen CPNs could be employed for highly effective PTT ablation of tumors, and in the meanwhile could be easily tracked by *in vivo* photoacoustic (PA) imaging, the latter of which could provide useful information of the tumor (*e.g.*, tumor size, location, and time-dependent uptake of Ru-Phen CPNs) to enable optimization of the therapeutic plan 35-37. Importantly, as observed by PA imaging and further verified by direct measurement of Ru^3+^ levels in organs and urine, Ru-Phen CPNs show efficient renal excretion after intravenous (i.v.) injection into mice without long-term retention and toxicity. Thus, our work presents safe and effective theranostics based on CPNs, promising for potential clinical translation.

## Experimental section

*Synthesis of Ru-Phen CPNs:* First, 1 mmol RuCl_3_ and 2 mmol Phen were mixed in 50 ml water under magnetic stirring, then heated to 120 °C for 6 hours in Teflon. The product was dialyzed against distilled water for 24 hours to remover free ions and molecules with a molecular weight cutoff (MWCO) of 3500 Da. After that, the solution was centrifugated at 8000 rpm for 5 min to remove large scaled impurities. The final solution was kept in dark for future use.

*Characterizations:* Transmission electron microscope (TEM) images and energy dispersive X-ray spectroscopy (EDS) were acquired by an FEI Tecnai F20 TEM at an acceleration voltage of 200 kV. X-ray photoelectron spectroscopy (XPS) measurement was conducted with a PHI Quantera SXM instrument equipped with an Al X-ray excitation source (1486.6 eV). Powder X-ray diffraction (XRD) spectra were collected on a PANalytical X-ray diffractometer. UV-Vis-NIR absorption spectra were performed with a PerkinElmer UV-Vis spectrophotometer. The dynamic light scattering (DLS) of CPNs was recorded using MALVERN ZEN3690. The absolute Ru contents were measured by ICP-MS (Inductively coupled plasma mass spectrometry).

*Calculation of the mass extinction coefficient:* To obtain 808 nm absorption capability of Ru-Phen CPNs, the mass extinction coefficient ε(λ) was calculated by the Lambert-Beer law according to the previous study: A(λ) = εLC. In this formula, at the wavelength of λ, A is the absorbance of Ru-Phen CPNs at the concentration of C in g/L. L is the pathlength which is 1 cm. At 808 nm, the mass extinction coefficient ε(λ) could be calculated to be 27 L⋅g^-1^⋅cm^-1^.

*Calculation of the photothermal conversion efficiency:* According to the previous references^41^, the 808 nm laser photothermal conversion efficiency (*η*) of Ru-Phen CPNs nanoparticles can be calculated to be 60.69 % (Detailed in the Supporting Information).

*Cellular experiments:* 4T1 murine breast cancer cells were used for *in vitro* cytotoxicity test and photothermal therapy. 4T1 cells were first cultured with different concentrations (C_Ru_ = 300, 150, 75, 38, 19, 10, 0 µg/ml) of Ru-Phen CPNs for 12 hours and the standard thiazolyl tetrazolium (MTT) assay was used for qualifying the relative viabilities of the cells.

For PTT, 4T1 cells were firstly cultured with different concentrations (C_Ru_ = 300, 150, 75, 38, 19, 10, 0 µg/ml) of Ru-Phen CPNs for 12 hours and then irradiated under a 808 nm laser at 0.5 W/cm^2^ for 10 minutes. Then the standard MTT assay was used for qualifying the relative viabilities of the cells. The cells could also be stained with calcein AM/propidium iodide (PI) after cultured with PBS and Ru-Phen CPNs (125 µg/ml) with and without 808 laser irradiations (0.5 W/cm^2^ for 10 minutes) then imaged under confocal fluorescence microscopy (Leica TCS-SP5II, Germany).

*Tumor model:* Balb/c mice were purchased from Suzhou Belda Biopharmaceutical Co. Ltd and treated following protocols approved by Soochow University Laboratory Animal Center. After the subcutaneous injection of 50 µl of 2*10^6^ 4T1 cells on the back of each female Balb/c mouse and when the tumor volume reached 100 mm^3^, the* in vivo* experiments were taken place.

*In vivo PA imaging:* After intravenously injected of Ru-Phen CPNs at the Ru dose of 2 mg/kg, the mice were imaged at different time points (0, 2, 4, 8, 12, 24 hours) by a Visualsonic Vevo 2100 LAZER system with an excitation wavelength at 808 nm. For *ex vivo* PA imaging of kidneys, the kidneys were collected at 120 min post i.v. injection of Ru-Phen and imaged with an excitation wavelength at 808 nm.

*In vivo PTT:* For *in vivo* PTT, 4T1 bearing mice randomly separated into four groups (n=5 per group): (1) PBS; (2) PBS + Laser; (3) Ru-Phen CPNs; (4) Ru-Phen CPNs + Laser. For group 3 and 4, Ru-Phen CPNs at the Ru dose of 2 mg/kg were intravenously injected. After 12 hours, the tumors in group 2 and 4 were exposed under 0.5 W/cm^2^ 808 nm laser for 10 minutes. The IR thermal images and the tumor temperature were taken by IR thermal camera (Fortric 225). Every two days after the PTT, the tumor size was measured by digital caliper and calculated by the following formula: tumor volume (V) = (tumor width)^2^ X (tumor length)/2.

*In vivo biodistribution study*: After various time points post *i.v.* injection of Ru-Phen CPNs at a Ru dose of 2 mg/kg, the main organs were collected from mice to analysis the Ru contents by ICP-MS.

*Hematology analysis:* After* i.v.* injection of Ru-Phen CPNs (2 mg/kg) for 1, 7, 14, and 30 days, the whole blood of mice were collected for blood biochemistry assay and complete blood panel analysis. The untreated mice were used for the control.

## Results and discussion

Ultra-small Ru-Phen CPNs were obtained by mixing RuCl_3_ and phenanthrolinemonohydrate (Phen) through a simple hydrothermal method (Experiment section). Phen was one of the simplest polypyridyl in which the nitrogen atoms could coordinate with Ru^3+^ ions. Thereafter, water-insoluble colorless Phen would be transformed into blue-colored Ru-Phen CPNs with great water solubility. As revealed by transmission electron microscopy (TEM), uniform nanodots with an average diameter of ~ 6.5 nm were formed (**Figure 1B-C**). From the high-resolution TEM image, the synthesized Ru-Phen CPNs showed low diffraction contrast without any obvious lattice fringes, indicating the amorphous structure for those formed nanodots, consistent to the result of X-ray diffraction (XRD, **Figure S1**). Importantly, without any surface modification, the as-synthesized ultra-small Ru-Phen CPNs appeared to be very stable in various physiological solutions and the hydrodynamic diameter of Ru-Phen CPNs was determined to be ~ 8 nm (**Figure 1D**). The presence of Ru in the Ru-Phen CPNs was also confirmed by energy-dispersive X-ray spectroscopy (EDX, **Figure 1E**) and X-ray photoelectron spectroscopy (XPS, **Figure 1F**). The two strong binding energy peaks at ~284.2 eV and ~280.7 eV were attributed to Ru 3d_3/2_ and Ru 3d_5/2_ of Ru, respectively. As monitored by the thermogravimetric analysis (TGA) and inductively coupled plasma mass spectrometry (ICP-MS), the molar ratio of Ru in such Ru-Phen CPNs was determined to be 2 : 1 (**Figure S2**). The infrared peaks of Ru-Phen CPNs at 1560-1620 cm^-1^ (the C=C and N=C stretching band) decreased and showed an obvious red-shift to longer wavelength, indicating that the nitrogen on Phen coordinated with Ru ions (**Figure S3**).

**Figure 2:**
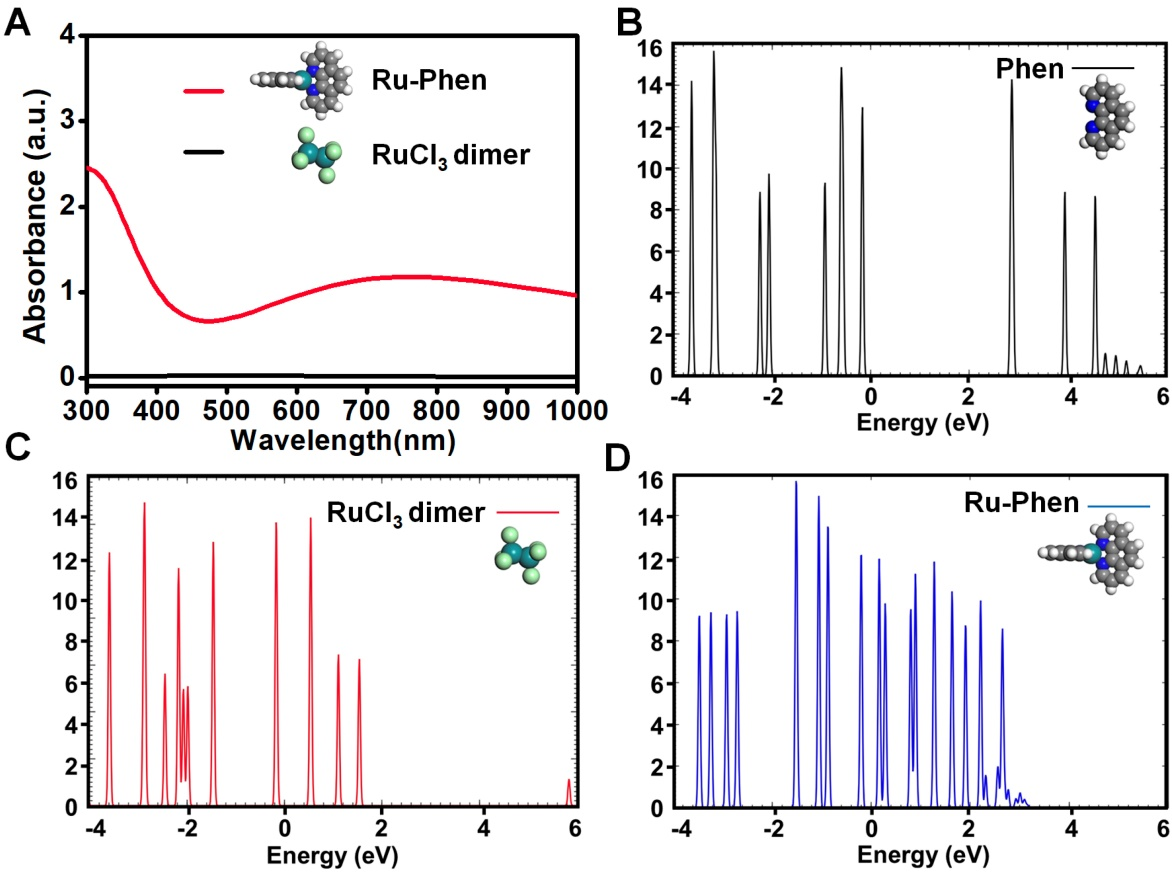
Theoretical calculation of Ru-Phen CPNs. (**A**) Theoretical calculation of UV-vis-NIR absorbance for Ru-Phen CPNs and RuCl_3_. The density of states (DOS) of (**B**) a Phen ligand, (**C**) the dimer of RuCl_3_ and (**D**) the Ru-Phen CPNs. The Fermi energy is zero eV.

Compared to RuCl_3_ solution, the UV-vis-NIR spectrum of Ru-Phen CPNs with deep blue solution showed superior high absorbance in a wide spectrum range from 700~1000 nm (**Figure 1G, Figure S4**). The mass extinction coefficient of Ru-Phen CPNs at 808 nm was calculated to be ~27 L⋅g^-1^⋅cm^-1^, which was among the top ones compared to the previously reported photothermal agents 38-42. Interestingly, the absorbance and the structure of the Ru-Phen CPNs were kept constant during the adjustment the reaction ratio of Ru to Phen (1 : 1, 2 : 1, and 5 : 1), probably due to the fixed coordination number of Phen molecule linked with Ru^3+^ (**Figure S5**). According to the theoretical calculation (**Figure 2A**), one Ru^3+^ ion was coordinated with two Phen molecules in aqueous solution to form the Ru-Phen CPNs with high absorbance. In order to reveal the physical insights of the enhanced NIR absorbance of the Ru-Phen CPNs, the density of states (DOS) of the isolated RuCl_3_ dimer, the Phen ligand, and the Ru-Phen CPNs were investigated. As seen in **Figure 2B-D**, the DOS near the Fermi energy (*e.g.*, -2.0 eV ~ 2.0 eV) had been dramatically increased when the ligands were coupled with RuCl_3_. This indicated that the energy gaps among front orbitals in the Ru-Phen CPNs were small, and of importance, there were enough empty orbitals available to accommodate the photo-excited electrons. Thus, when the electrons were excited with photo energy, the hoping of electrons in the front orbitals were much more feasible in Ru-Phen CPNs, resulting in an increase of the NIR absorbance.

To evaluate the stability of Ru-Phen CPNs, the UV-vis-NIR absorbance and hydrodynamic size were recorded as shown in **Figure S6-S7**. Even after 7 days dispersed in water (pH=7.4, pH=6.5, and pH=5.5), PBS, or medium (1640 cell medium + 10 % FBS), the UV-vis-NIR absorbance and hydrodynamic size were kept the same. Moreover, the Ru-Phen CPNs were further processed the freeze-drying for one month and then re-dispersion, no unexpected UV-vis-NIR absorbance or hydrodynamic size was shown (**Figure S8**). This result strongly proved the good stability of the synthesized Ru-Phen CPNs and encouraged the further application of their properties.

**Figure 3:**
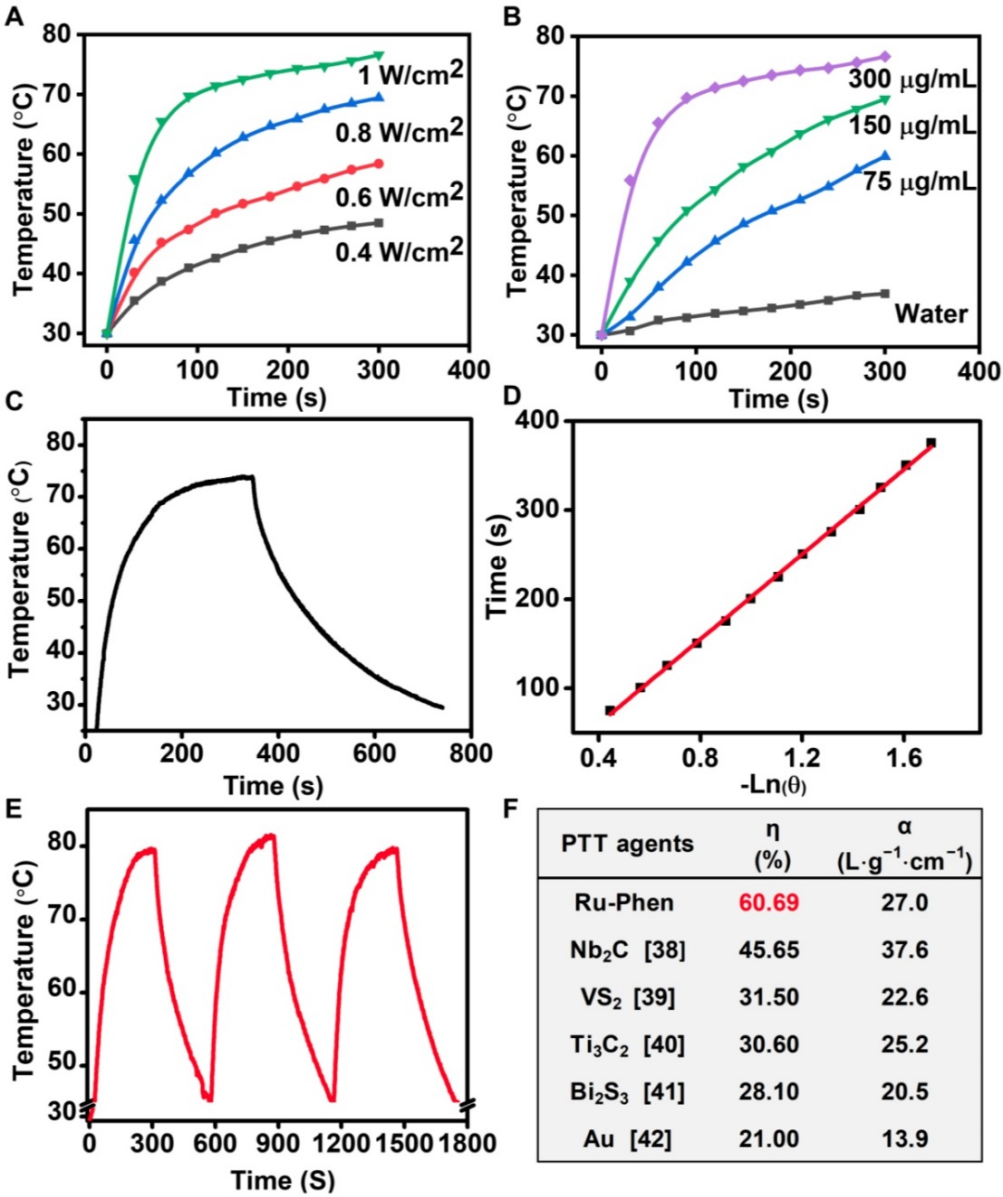
Photothermal conversion effect of Ru-Phen CPNs. Photothermal effect of Ru-Phen CPNs irradiated by 808-nm laser under (**A&B**) different power densities (1, 0.8, 0.6, and 0.4 W/cm^2^, with the concentration of 300 μg/mL) and concentrations (300, 150, 75 and 0 μg/mL, with the power density of 1 W/cm^2^). (**C**) Photothermal effect of Ru-Phen CPNs under irradiation of the NIR laser (808 nm, 1 W/cm^2^, 5 min) and thereafter laser shut-off. (**D**) the time constant (τs) for the heat transfer from the system determined by applying the linear time data from the cooling period. (**E**) Recycling heating profile of Ru-Phen CPNs under the 808 nm laser irradiation (1 W/cm^2^) for three on/off cycles. (**F**) The photothermal conversion efficiency (η) and mass extinction coefficient (α, unit: L⋅g^-1^⋅cm^-1^) of different phothothermal therapy (PTT) agents at 808 nm, references 38-42.

Taking advantage of their high NIR absorbance, Ru-Phen CPNs could be employed as a photothermal agent. Under 808-nm laser irradiation, Ru-Phen CPNs showed a significant temperature increment, and the raised temperature was dependent on the increased power density and concentration (**Figure 3A-B**, **Figure S9**). Meantime, the photothermal conversion efficiency, which indicated the capability of photothermal agents to transfer laser energy into heat, was determined to be ~ 60.69 % (**Figure 3C-D**), much higher than the previously reported photothermal agents (**Figure 3F**). Importantly, the Ru-Phen CPNs also possessed excellent photothermal stability after three cycles ON/OFF laser irradiation evidenced by the changeless UV-vis-NIR absorbance and photothermal effect (**Figure 3E, Figure S1, S10**). Moreover, the XRD pattern of Ru-Phen CPNs showed no obvious variation after the photothermal process owing to their excellent photothermal stability.

**Figure 4:**
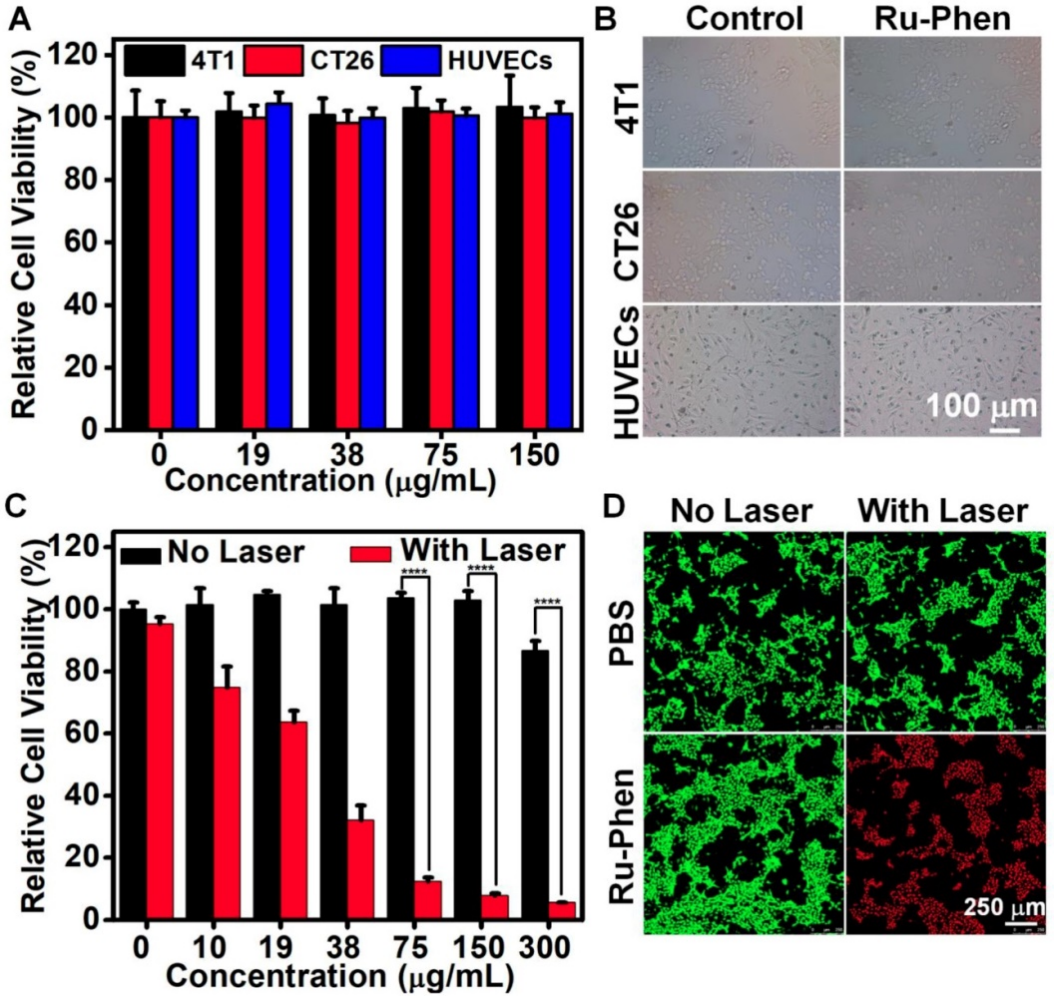
*In vitro* PTT. (**A**) Relative cell viabilities and (**B**) optical photos of 4T1, CT 26 and HUVECs after cultured with Ru-Phen CPNs for 24 hours. (**C**) Relative cell viabilities of 4T1 treated by Ru-Phen CPNs under the irradiation (808 nm, 0.5 W/cm^2^, 10 min). (**D**) The Calcein AM/propidium iodide (PI) co-stained images of 4T1 cells incubated with Ru-Phen CPNs after laser irradiation (808 nm, 0.5 W/cm^2^ 10 min). P values were calculated based on Tukey's post-test (****p < 0.0001).

Inspired by the superb intrinsic optical property of Ru-Phen CPNs, we tried to use them as the photothermal agent for cancer therapy. First of all, the cell uptake of Ru-Phen CPNs was evaluated. After incubating 4T1 murine breast cancer cells with Ru-Phen CPNs for different times, the contents of Ru-Phen CPNs in cells was tested by ICP-MS. As shown in **Figure S11**, an accumulated Ru content in cells was detected and proved the successful entry of the CPNs in cells. Next, the cytotoxicity of Ru-Phen CPNs was investigated. The 4T1 cells, CT26 murine adenocarcinoma cells, and Human umbilical vein endothelial cells (HUVECs) were incubated with various concentrations of Ru-Phen CPNs for different times. The cell viability of all the three kinds of cells didn't change with the increased Ru-Phen CPNs concentration and always kept entire (**Figure 4A, Figure S12**). In addition, even under the high concentration of 150 μg/mL, neither of them showed abnormal morphology change (**Figure 4B**, **Figure S13**). These results strongly evidenced the negligible cytotoxicity of Ru-Phen CPNs. However, under the laser irradiation, the cell viability decreased dramatically with the increase of CPNs concentrations (**Figure 4C**). Following the laser irradiation, the cells were further checked by co-staining with calcein AM (AM, live cells) and propidium iodide (PI, dead cells). Almost all the cells incubated with Ru-Phen CPNs were killed under the laser irradiation (**Figure 4D**), demonstrating the superb photothermal effect of the synthesized Ru-Phen CPNs.

**Figure 5:**
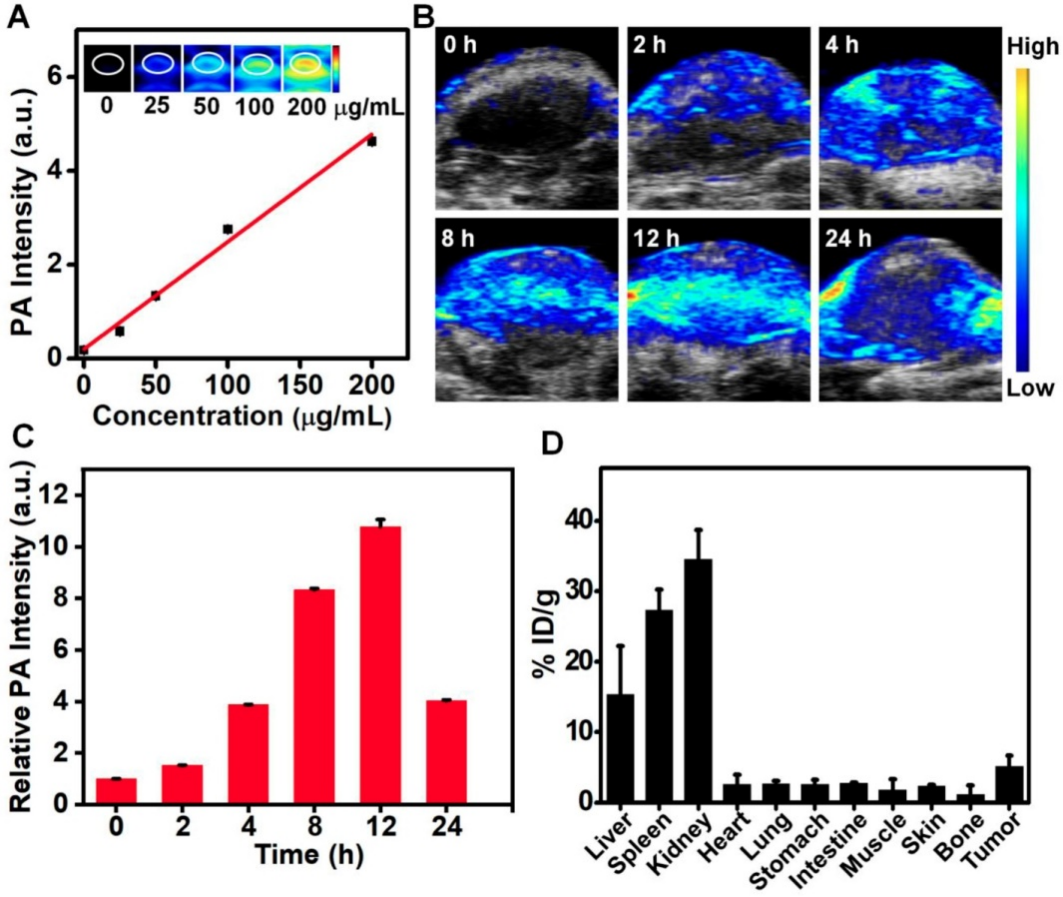
*In vivo* PA imaging and biodistribution. (**A-C**) PA imaging of Ru-Phen CPNs. (**A**) PA images and intensity of Ru-Phen CPNs at different concentrations. (**B**) PA images of tumors on 4T1 tumor-bearing mice post *i.v.* injection at different time points. (**C**) The PA signal of tumor at different time points. (**D**) The biodistribution of Ru-Phen CPNs measured by ICP-MS at 12 h *p.i.*.

Utilizing the strong absorbance and photothermal conversion efficiency of the synthesized Ru-Phen CPNs, they also could be used for PA imaging, which had been wildly employed in medical detection and imaging with deeper tissue penetration and higher resolution compared to the conventional fluorescence imaging. The PA signals were linearly dependent on the concentrations of Ru-Phen CPNs. However, the two other compounds, Phen and RuCl_3_ didn't show any PA response due to the low absorbance (**Figure 5A, Figure S14**). After i.v. injection of Ru-Phen CPNs into 4T1 tumor-bearing mice, the PA signals in the tumor appeared gradually, indicating that the time-dependent accumulation of Ru-Phen CPNs in the tumor by the enhanced permeability and retention (EPR) effect (**Figure 5B**). The tumor PA signals reached the maximum at 12 h (11-fold enhancement) and then decreased over time (5-fold enhancement at 24 h), indicating the gradual clearance of Ru-Phen CPNs from the body due to their ultra-small size (**Figure 5C-D**). Moreover, as one of the most important indicators of *in vivo* behavior of nanomaterials, the time-dependent blood circulation of Ru-Phen CPNs was also investigated. After intravenously injected with Ru-Phen CPNs, the blood of treated mice was taken out and weighted at different time points for ICP-MS to quantify the content of Ru-Phen CPNs. As it was shown in **Figure S15**, the long-time blood circulation half-life was determined to be 1.09 ± 0.11 hs for the first phase and 13.86 ± 1.77 hs for the second phase.

**Figure 6:**
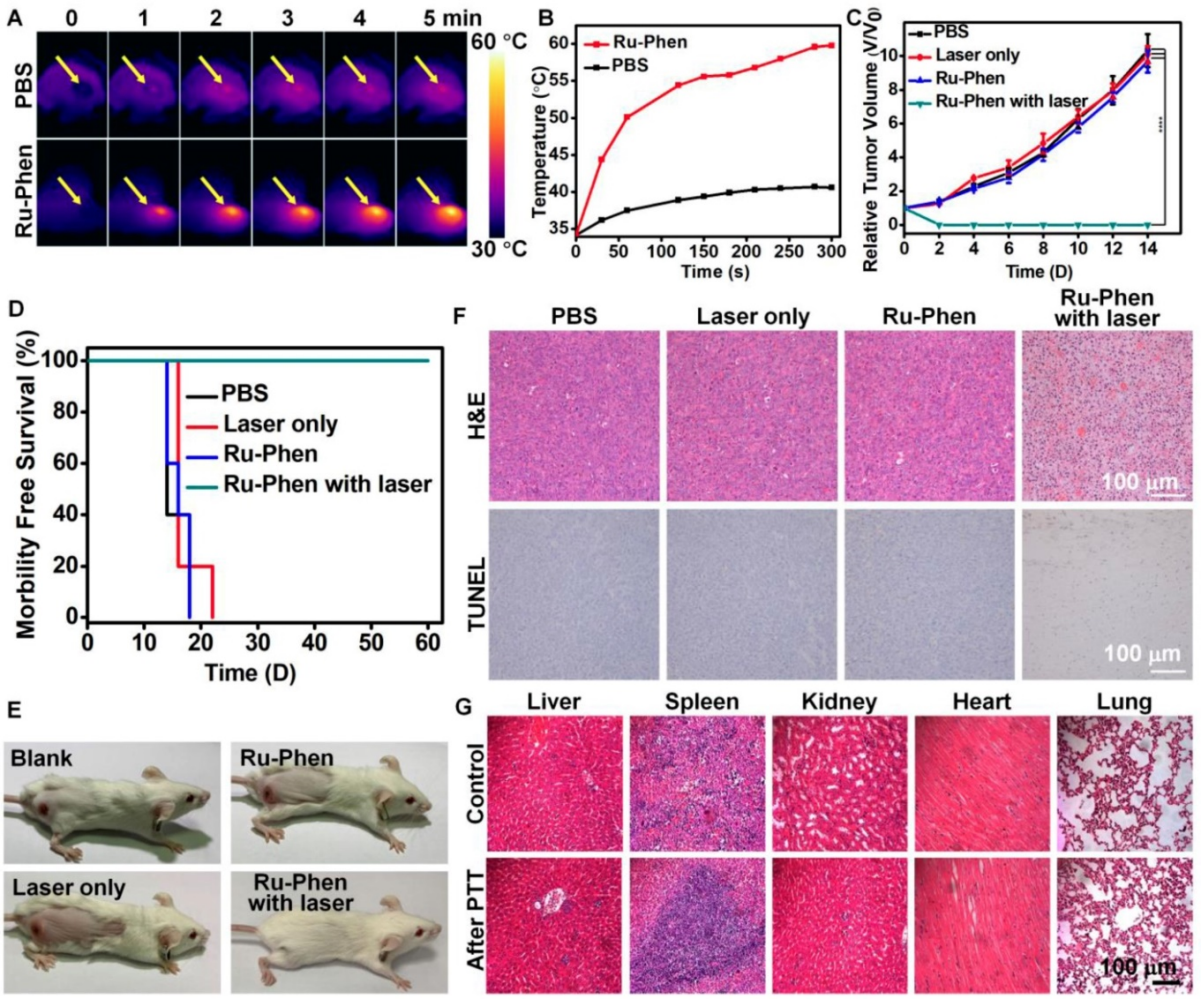
*In vivo* PTT. IR thermal imaging (**A**) and corresponding tumor temperature changes (**B**) of 4T1tumor bearing mice with *i.v.* injection of Ru-Phen CPNs under laser irradiation (808 nm, 0.5 W/cm^2^, 5 min). (**C**) The growth of 4T1 tumors in different groups of mice after various treatments. (**D**) Survival curves of mice after various treatments. (**E**) Optical photos of tumor-bearing mice treated with/without Ru-Phen CPNs and laser irradiation (808 nm, 0.5 W/cm^2^, 5 min). (**F**) H&E and TUNEL stained tumor sections and (**G**) H&E stained tissue sections of major organs of mice after PTT with Ru-Phen CPNs injection 12 h *p.i.* (injection dose: Ru=2 mg/kg).

According to PA imaging, PTT was carried out at 12 h post i.v. injection of Ru-Phen CPNs into the tumor-bearing mice. The temperature of the tumor was increased to 60 ^o^C within 5 min under irradiation by an 808-nm laser (0.5 W/cm^2^) as monitored by the IR thermal imaging (**Figure 6A-B**), in marked contrast to the no significant tumor temperature change on mice injected with saline under the same irradiation condition. We next examined the photothermal therapeutic effect of Ru-Phen CPNs. The tumor growth and body weight were monitored every the other day. Remarkably, we found that the mice after PTT with Ru-Phen CPNs became tumor-freeand survived for over two months (**Figure 6E**). However, the mice in the other three groups all died in 18-22 days owing to the rapid tumor growth (**Figure 6C-D, Figure S16**). In the meantime, the hematoxylin & eosin (H&E) staining and TdT-mediated dUTP nick-end labeling (TUNEL) staining of tumor were also carried out to analyze the tumor damage after different treatments. From **Figure 6F**, after treated with Ru-Phen CPNs and NIR irradiation, the tumor tissue received the fatal hyperthermia and suffered complete apoptosis compared with the other three groups. These results strongly demonstrated the high efficiency of Ru-Phen CPNs enhanced PTT. After that, the main organs (liver, spleen, kidney, heart, and lung) were taken out for H&E staining (**Figure 6G**), no appreciable abnormality in these organs was observed. Our results demonstrated that PTT with Ru-Phen CPNs could completely destruct tumor without any toxic effect.

**Figure 7:**
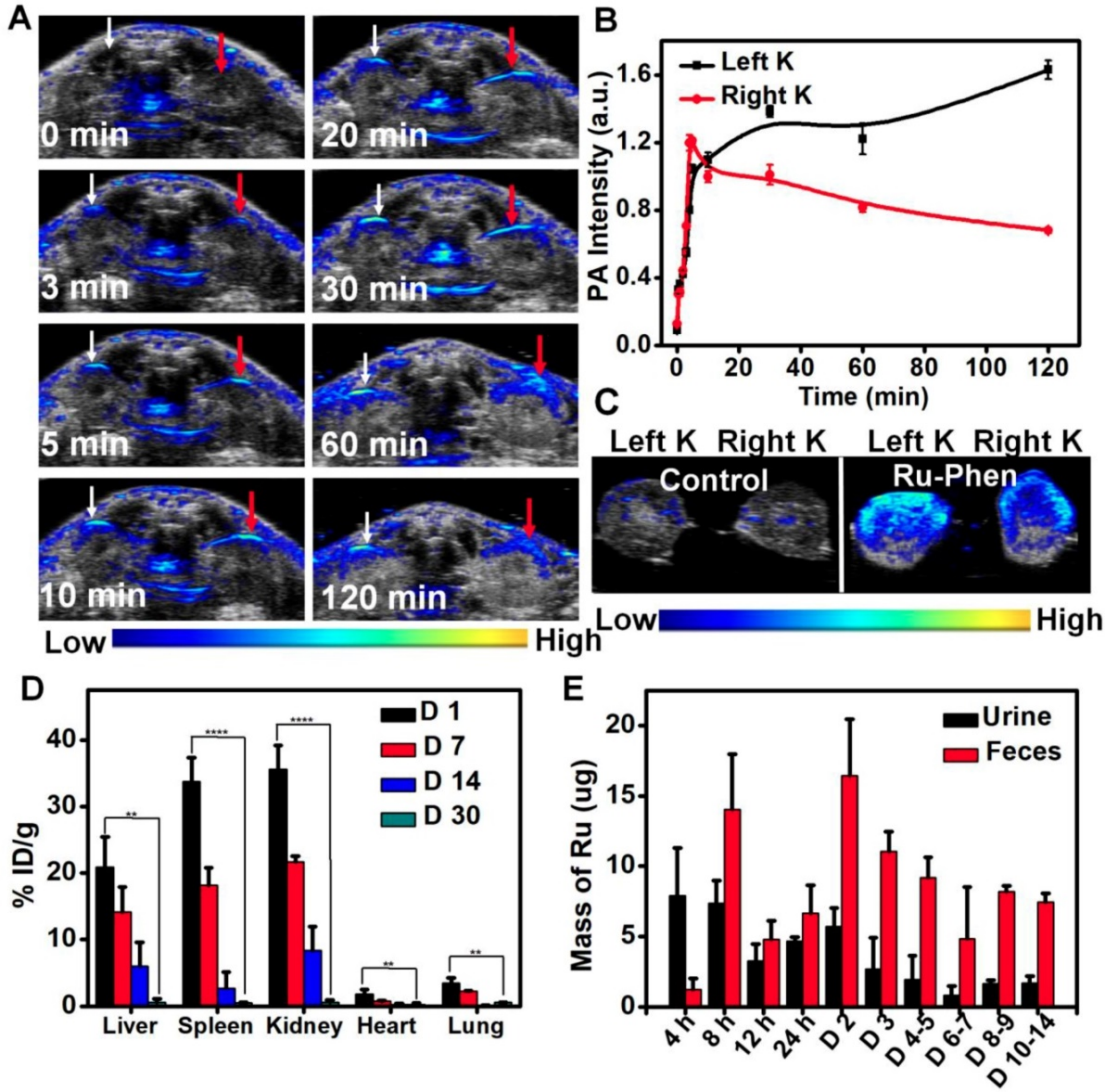
*In vivo* clearance behavior of Ru-Phen CPNs. (**A-C**) *In vivo* and *ex vivo* PA imaging of kidneys. (**A**) PA images of kidneys of mice at different time points post *i.v.* injection of Ru-Phen CPNs. White arrow: Left kidney; Red arrow: Right Kidney. (**B**) The relative PA signal of kidneys post injection of Ru-Phen CPNs at different time points. (**C**) PA images of kidneys of the mouse at 2 h post *i.v.* injection of Ru-Phen CPNs. K: Kidney. (**D-E**) Biodegradation of Ru-Phen CPNs. (**D**) The biodistribution of Ru-Phen CPNs in mice at 1, 7, 14, and 30 days post *i.v.* injection of Ru-Phen CPNs. The Ru contents were measured by ICP-MS. (**E**) The detected Ru mass in feces and urine at different time points post *i.v.* injection of Ru-Phen CPNs. (Injection dose: Ru=7 mg/kg).

Considering the ultra-small size of Ru-Phen CPNs, we wondered whether they could be excreted out of the body by urine, the fastest body clearance pathway. PA imaging was used to real-time monitor the renal clearance behavior of the Ru-Phen CPNs by detecting signals in kidneys. After i.v. injection of Ru-Phen CPNs, the PA signals in the kidneys quickly showed up, indicating the accumulation of Ru-Phen CPNs in the kidneys (**Figure 7A-B**). Furthermore, the kidneys were collected at 2 h post-injection of Ru-Phen CPNs for *ex vivo* PA imaging. Strong PA signals from Ru-Phen were also observed in those kidneys, suggesting the possible renal excretion of those CPNs with ultra-small sizes (**Figure 7C**). To further study the clearance behaviors of CPNs, the Ru contents in main organs at different time points post-injection was also determined by ICP-MS. High concentrations of CPNs in kidneys indicated the existence of the Ru-Phen CPNs for further urine clearance. Notably, rather low levels of Ru^3+^ could be observed in all examined organs of mice (**Figure 7D-E**), demonstrating that most of Ru-Phen CPNs were excreted from the body.

Furthermore, we also collected the excretion of mice after i.v. injection of Ru-Phen CPNs and analyzed the Ru contents in CPNs by ICP-MS. It could be found that partial of the Ru-Phen CPNs were excreted by urine through kidneys in a short time period due to their ultra-small size, and those accumulated in the reticuloendothelial system (RES) including liver and spleen could be further cleared from the body via feces. Therefore, nearly 86.95 ± 6.72 % of total Ru-Phen CPNs was eliminated out from the body within two weeks. Therefore, the possibility of using PA imaging to monitor the renal clearance of the Ru-Phen CPNs had been successfully confirmed, as identified by ICP-MS quantification. At last, as evidenced by the hematology assay and H&E staining of the main organs at different time points post-injection (**Figure S17-18**), our Ru-Phen CPNs showed no obvious *in vivo* toxicity to mice at the tested dose, indicating the excellent bio-safety of the Ru-Phen CPNs.

## Conclusion

In summary, ultra-small Ru-Phen CPNs are synthesized for PA imaging-guided PTT. After coordination of Ru^3+^ and Phen, the obtained Ru-Phen CPNs show excellent water stability, biocompatibility, high NIR absorbance, and extraordinarily high photothermal conversion efficiency. With time-dependent tumor accumulation as revealed by *in vivo* PA imaging, such Ru-Phen CPNs could be employed for highly effective photothermal ablation of tumors. Moreover, benefited from the ultra-small size (~ 6.5 nm), the Ru-Phen CPNs could undergo fast renal clearance as monitored by PA imaging of kidneys and further confirmed by ICP-MS measurement. With little long-term body retention and no obvious *in vivo* toxicity, the Ru-Phen CPNs may be a promising type of photo-therapeutic agent with high and safe performance in imaging-guided cancer PTT.

## Supplementary Material

Supplementary information and figures.
