# Supplementary material for: Renal Clearable Ru-based Coordination Polymer Nanodots for Photoacoustic Imaging Guided Cancer Therapy
Source: Theranostics. 2019 Oct 21;9(26):8266–76. doi: 10.7150/thno.36986 (PMC6857064; doi:10.7150/thno.36986)
Supplement: Supplementary file 1 — Supplementary information and figures. [file thnov09p8266s1.pdf]

Supporting Information

Renal Clearable Ru-based Coordination Polymer Nanodots for Photoacoustic Imaging Guided Cancer Therapy

Rui Zhang, Xing Fan, Zhouqi Meng, Haiping Lin, Qiutong Jin, Fei Gong, Ziliang Dong, Youyong Li, Qian Chen, Zhuang Liu, and Liang Cheng*

Institute of Functional Nano & Soft Materials (FUNSOM), Jiangsu Key Laboratory for Carbon-Based Functional Materials and Devices, Soochow University, Suzhou 215123, China.

Email: lcheng2@suda.edu.cn

Calculation of the photothermal conversion efficiency*:* According to the pervious references[1-3], the whole energy balance of the entire system is

$$\sum_i m_i C_{p,i} \frac{dT}{dt} = Q_{Ru-Phen\ CPN} + Q_{Dis} - Q_{Surr}$$

in which m and C_p are the mass and heat capacity of water, T is the temperature of the solution, $Q_{Ru\text{-}Phen\ CPN}$ is the inputted energy of Ru-Phen CPN, Q_{Dis} is the inputted baseline energy of the sample cell and Q_{Surr} is the heat conducted away from the system surface to the environment by the air.

The 808 nm laser induced source term, $Q_{Ru\text{-}Phen\ CPN}$, represents heat dissipated by electron-phonon relaxation of the plasmon on the Ru-Phen CPN surface under the irradiation of 808 nm laser:

$$Q_{Ru-Phen\ CPN} = I(1 - 10^{-A_{808}})\eta \qquad (2)$$

Where I is incident laser power (in unit of mW), A_{808} is the absorbance of the Ru-Phen CPNs at wavelength of 808 nm, and η is the photothermal conversion efficiency from incident laser energy to thermal energy. In addition, Q_{Dis} expresses heat dissipated from light absorbed by the quartz cuvette sample cell itself, and it was measured independently using a sample cell containing pure water without Ru-Phen CPNs. $Q_{Ru\text{-}Phen\ CPN}$ is linear with temperature for the outgoing thermal energy, as the following equation:

$$Q_{Surr} = hS(T - T_{Surr}) \qquad (3)$$

where h is heat transfer coefficient, S is the surface area of the container, and T_{Surr} is ambient temperature of the surroundings.

Once the laser power is defined, the heat input ($Q_{Ru\text{-}Phen\ CPN}$ + Q_{Dis})will be finite. Since the heat output (Q_{Surr}) is increased along with the increase of the temperature according to the equation (3), the system temperature will rise to a maximum when the heat input is equal to heat output:

$$Q_{Ru-Phen\ CPNs} + Q_{Dis} = Q_{Surr-Max} = hS(T_{Max} - T_{Surr}) \qquad (4)$$

where $Q_{Surr\text{-}Max}$ is heat conduction away from the system surface by air when the sample cell reaches the equilibrium temperature, and T_{Max} is the equilibrium temperature. The 808 nm laser photothermal conversion efficiency (η) can be determined by substituting equation (2) for $Q_{Ru\text{-}Phen\ CPN}$ into equation (4) and rearranging to get

$$\eta = \frac{hS(T_{Max} - T_{Surr}) - Q_{Dis}}{I(1 - 10^{-A_{808}})} \quad (5)$$

where Q_{Dis} was measured independently to be 14 mW, the (T_{Max} - T_{surr}) was 43.8 °C, I is 1250 mW, A_{808} is the absorbance (5) of Ru-Phen CPNs at 808 nm. Thus, only the hS remains unknown for calculating η.

In order to get the hS, a dimensionless driving force temperature, θ is expressed using the maximum system temperature, T_{Max}

$$\theta = \frac{T - T_{Surr}}{T_{Max} - T_{Surr}} \quad (6)$$

and a sample system time constant τ_s

$$\tau_s = \frac{\sum_i m_i C_{p,i}}{hS} \quad (7)$$

which is substituted into equation (1) and rearranged to yield

$$\frac{d\theta}{dt} = \frac{1}{\tau_s}\left[\frac{Q_{Ru-Phen\ CPNs} + Q_{Dis}}{hS(T_{Max} - T_{Surr})} - \theta\right] \quad (8)$$

At the cooling period of Ru-Phen CPNs aqueous dispersion, the light source was shut off, the $Q_{Ru\text{-}Phen\ CPN} + Q_{Dis}$ =0, reducing the following equation

$$dt = -\tau_s \frac{d\theta}{\theta} \quad (9)$$

and integrating, giving the expression

$$t = -\tau_s \ln \theta \quad (10)$$

Therefore, time constant for heat transfer from the system of determined to be τ_s= 238.10 s by applying the linear time data from the cooling stage (300 s) *versus* negative natural logarithm of driving force temperature. In addition, the m is 1 g and the C is 4.2 J/g. Thus, according to equation (7), the hS is deduced to be 17.6396 mW/°C. Substituting this value of hS into equation (5), the 808 nm laser photothermal conversion efficiency (η) of Ru-Phen CPNs nanoparticles can be calculated to be 60.69 %.

Reference

[1] Roper DK, Ahn W, Hoepfner M. Microscale heat transfer transduced by surface plasmon resonant gold nanoparticles. J Phys Chem C. 2007; 111: 3636-41.

[2] Chen Y, Cheng L, Dong Z, Chao Y, Lei H, Zhao H, et al. Degradable vanadium disulfide nanostructures with unique optical and magnetic functions for cancer theranostics. Angew Chem Int Edit Engl. 2017; 56: 12991-6.

[3] Lin H, Wang X, Yu L, Chen Y, Shi J. Two-dimensional ultrathin MXene ceramic nanosheets for photothermal conversion. Nano Lett. 2017; 17: 384-91.

Supporting Figures

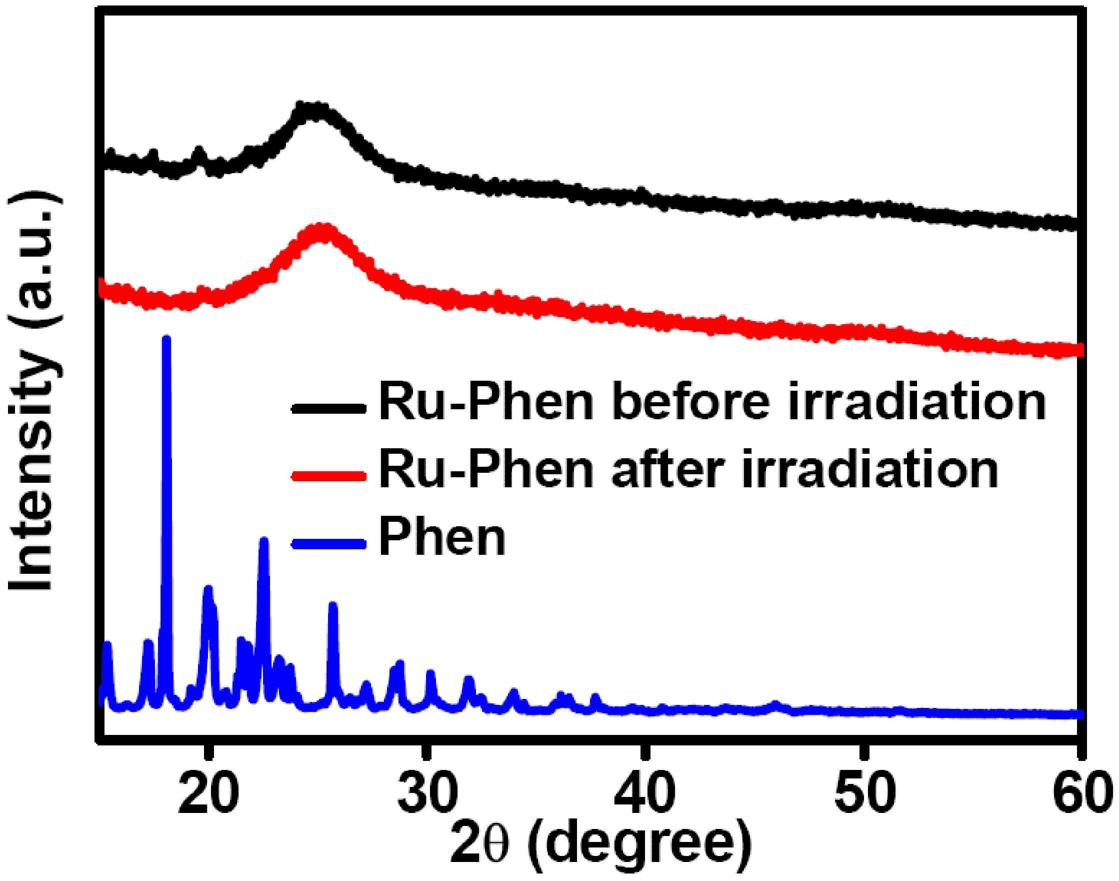

Figure S1. XRD data of Phen and Ru-Phen CPNs before/after irradiation.

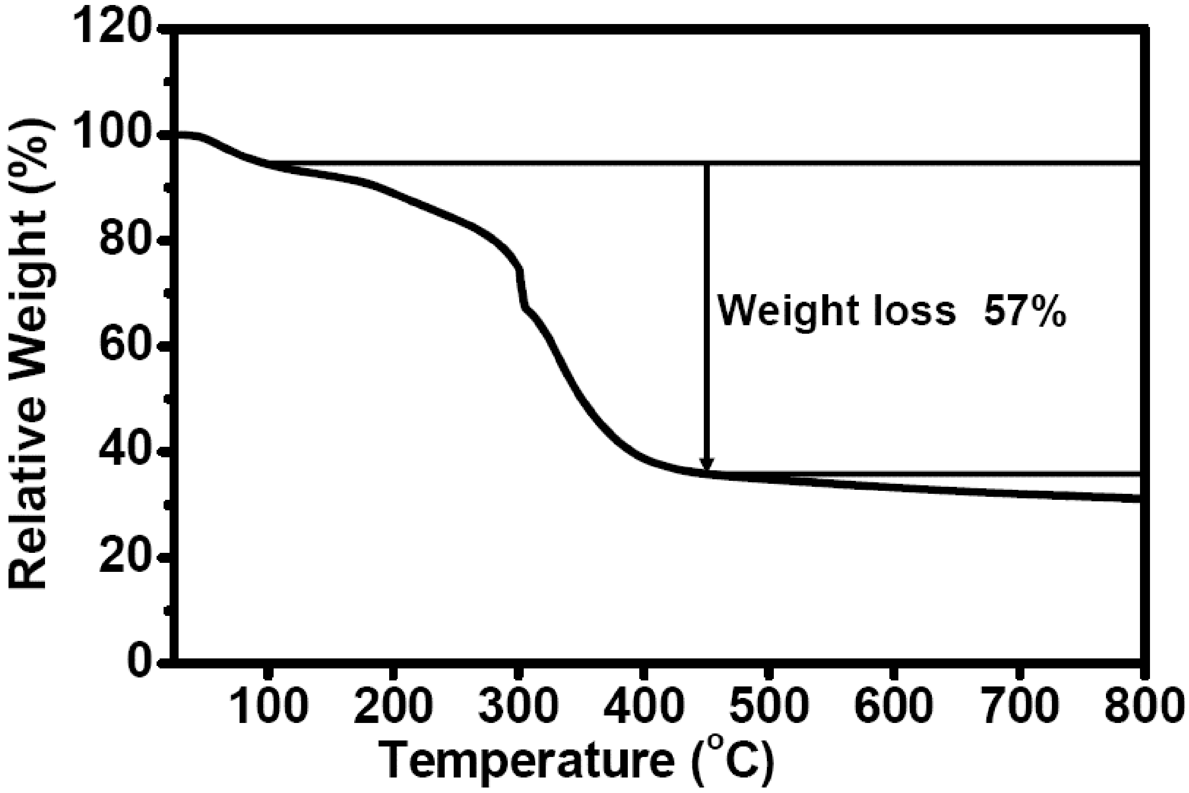

Figure S2. TGA analysis of the Ru-Phen CPNs.

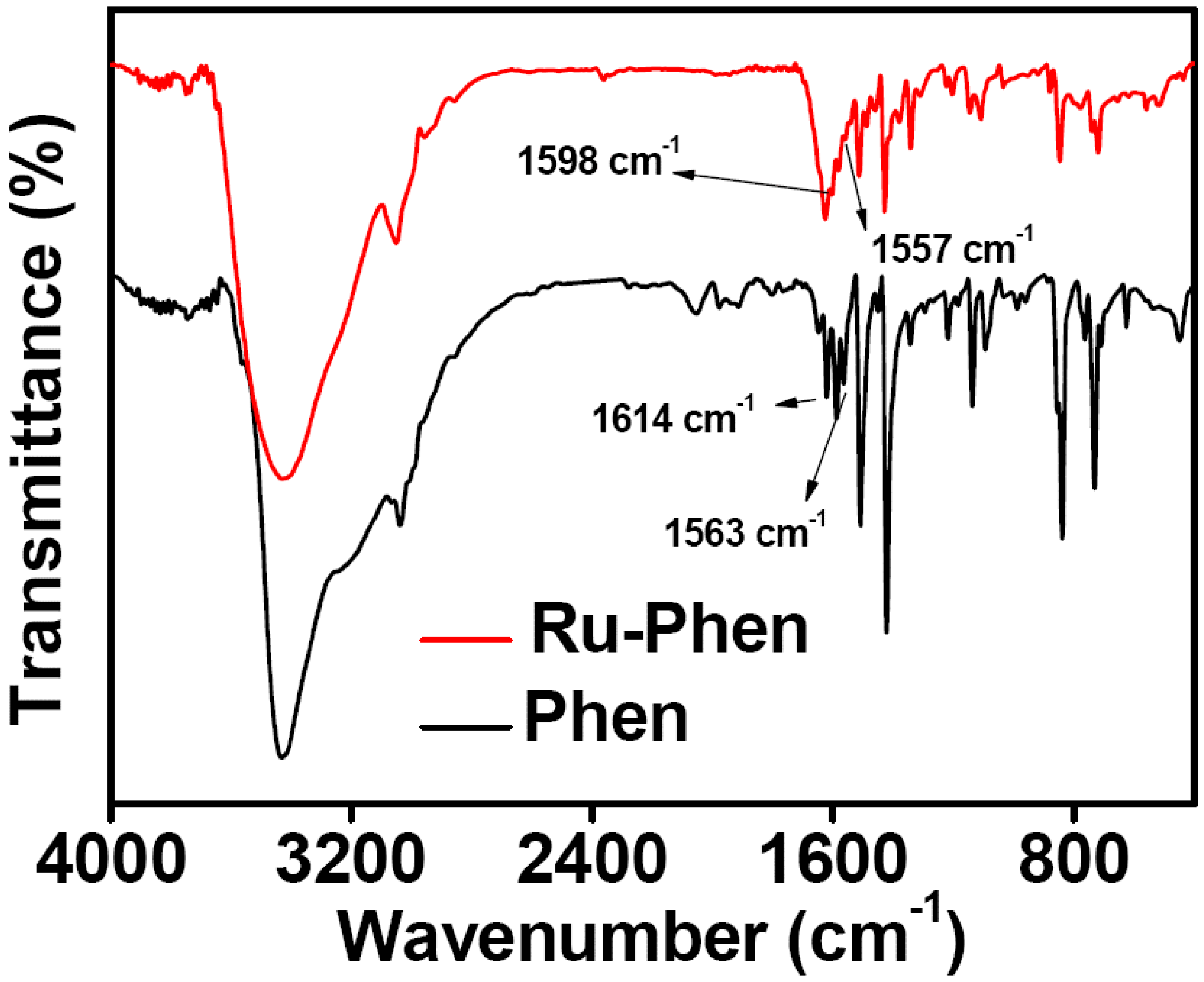

Figure S3. FTIR spectra of Ru-Phen CPNs and Phen molecule

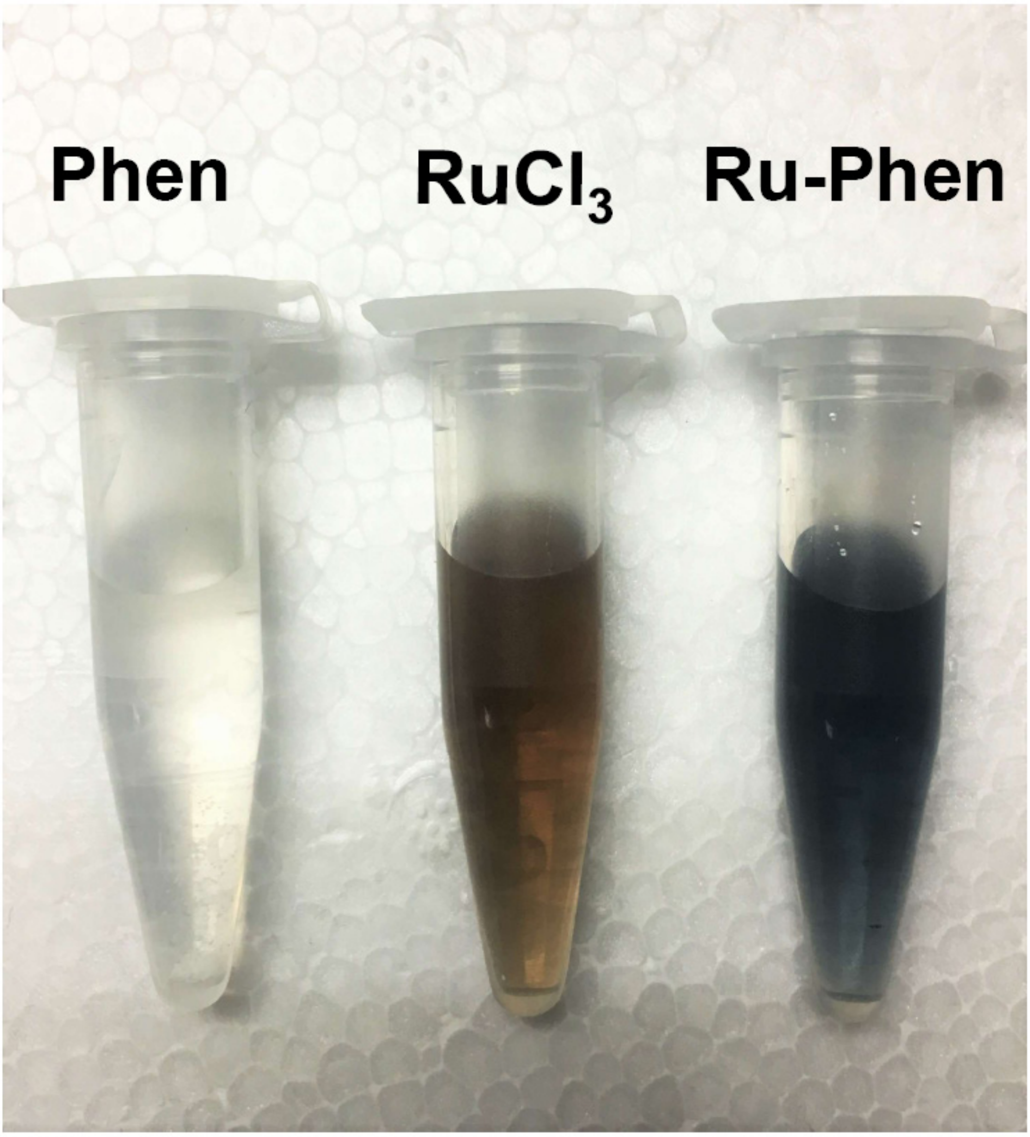

Figure S4. Optical image of Phen, $RuCl_3$, and Ru-Phen CPNs in water.

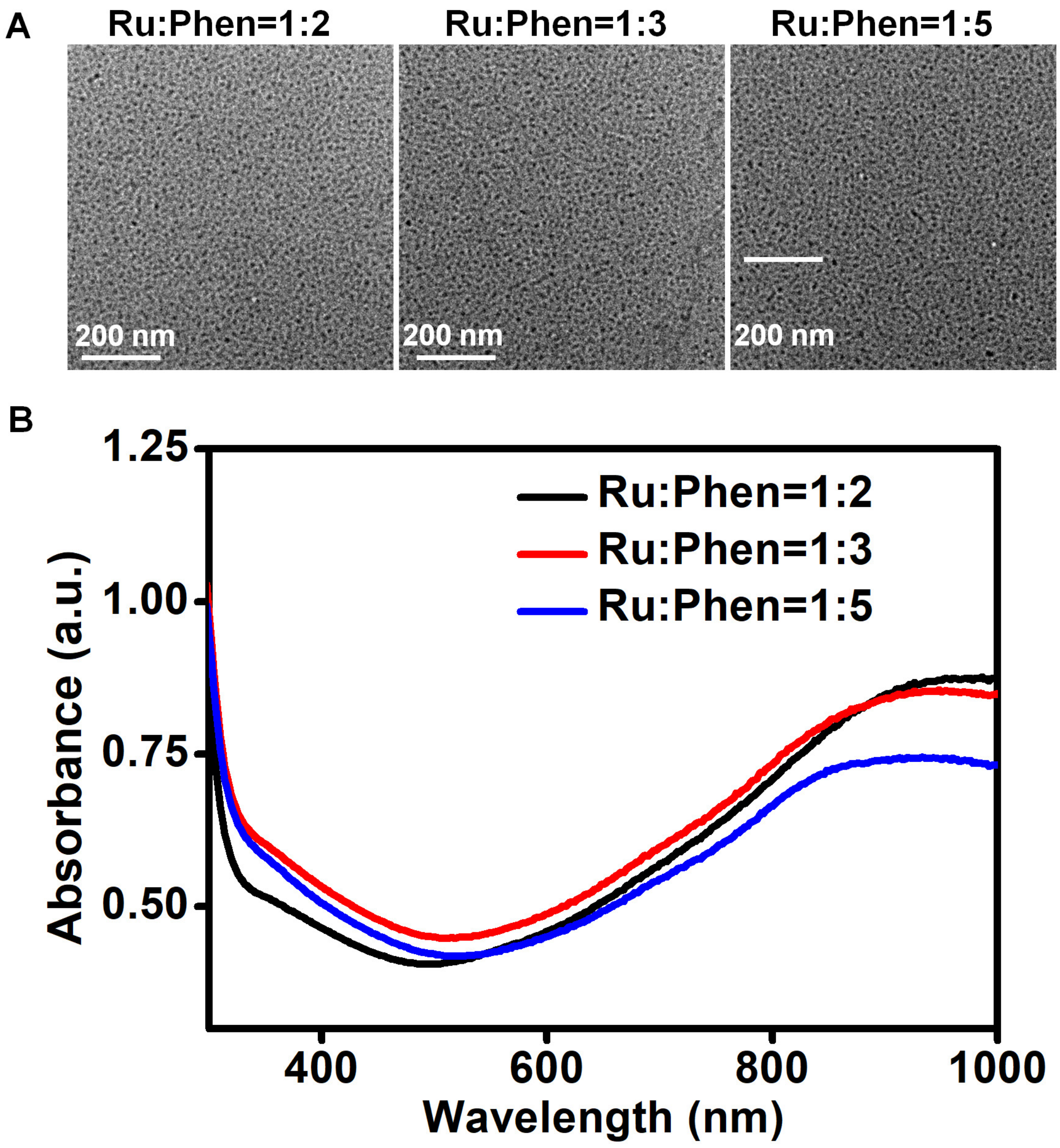

Figure S5. TEM images (**A**) and UV-vis-NIR spectra (**B**) of Ru-Phen CPNs with different reaction ratios of Ru to Phen (1:2, 1:3, and 1:5).

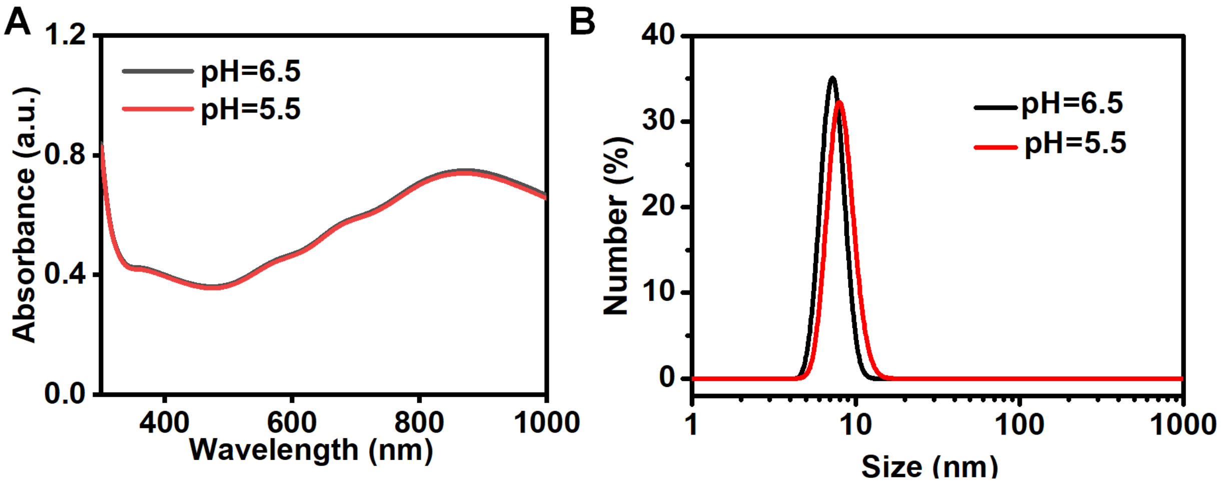

Figure S6. UV-vis-NIR absorbance (**A**) and hydrodynamic size (**B**) of Ru-Phen CPNs in different solutions (pH 6.5 and pH 5.5).

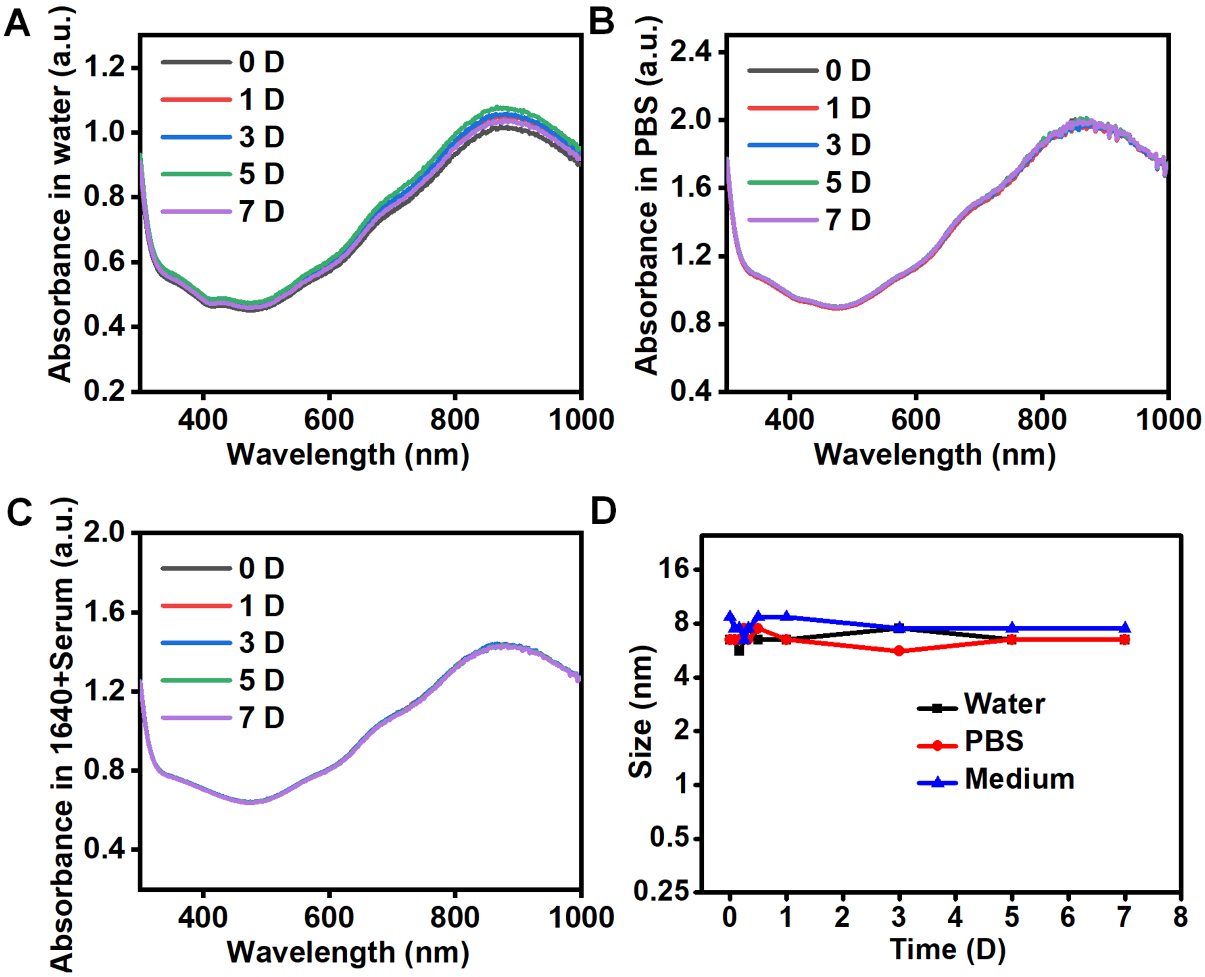

Figure S7. UV-vis-NIR absorbance of Ru-Phen CPNs in water (**A**), PBS (**B**), and medium (**C**) (1640 cell medium + 10 % FBS) at different time points (0, 1 D, 3 D, 5 D, and 7D). (**D**) Hydrodynamic size of Ru-Phen CPNs in water, PBS, and medium (1640 cell medium + 10 % FBS) at different time points (0, 2 h, 4 h, 6 h, 8 h, 12 h, 1 D, 3 D, 5 D, and 7D).

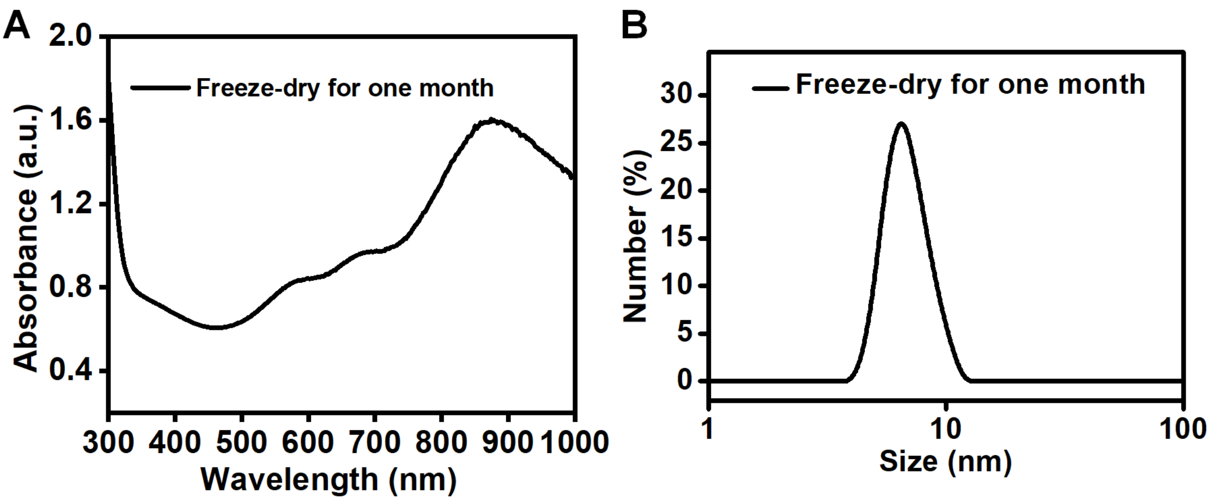

Figure S8. UV-vis-NIR absorbance (**A**) and hydrodynamic size (**B**) of Ru-Phen CPNs after freeze-dry and redissolution in water.

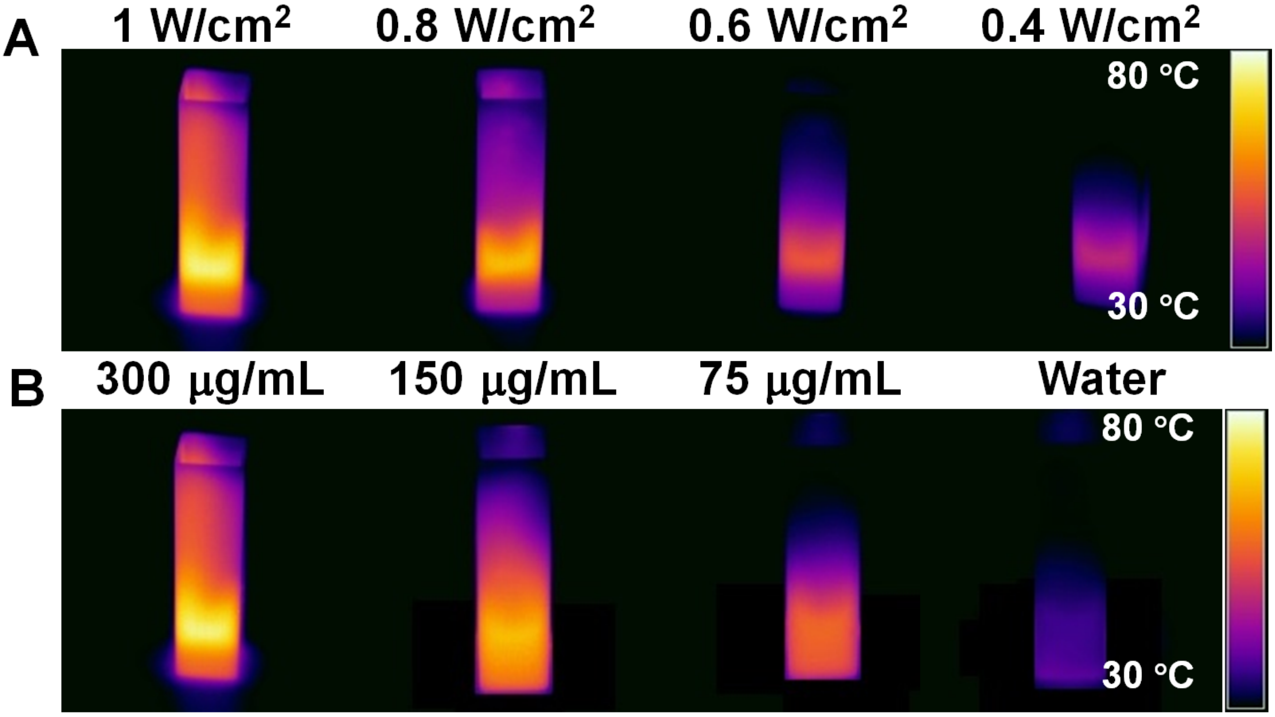

Figure S9. Thermal images of Ru-Phen CPNs irradiated by 808-nm laser under (**A**) different power densities (1, 0.8, 0.6, and 0.4 W/cm^2, with the concentration of 300 μg/mL,) and (**B**) concentrations (300, 150, 75, and 0 μg/mL, with the power densities of 1 W/cm^2).

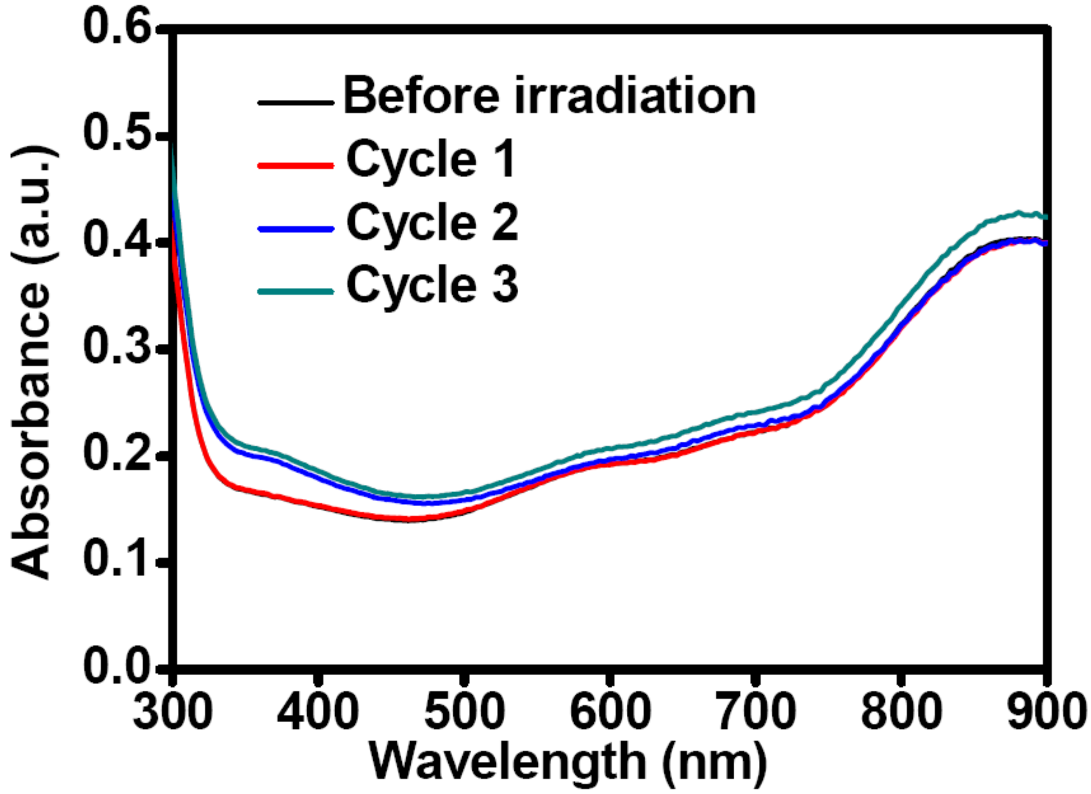

Figure S10. UV-vis-NIR spectra of Ru-Phen CPNs after three cycles of laser irradiation.

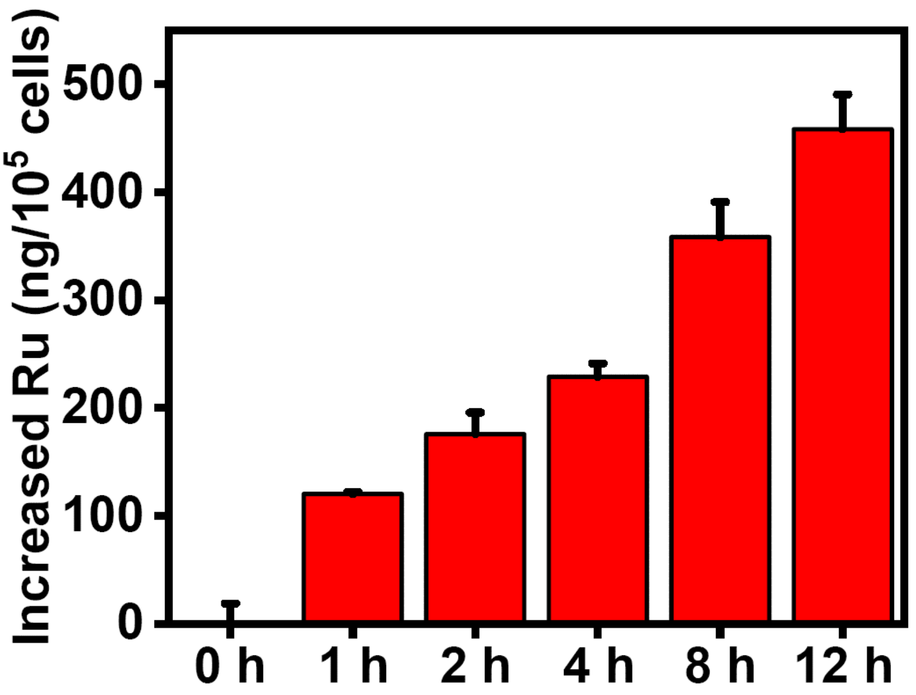

Figure S11. The accumulation of Ru-Phen CPNs in 4T1 cells at different time points.

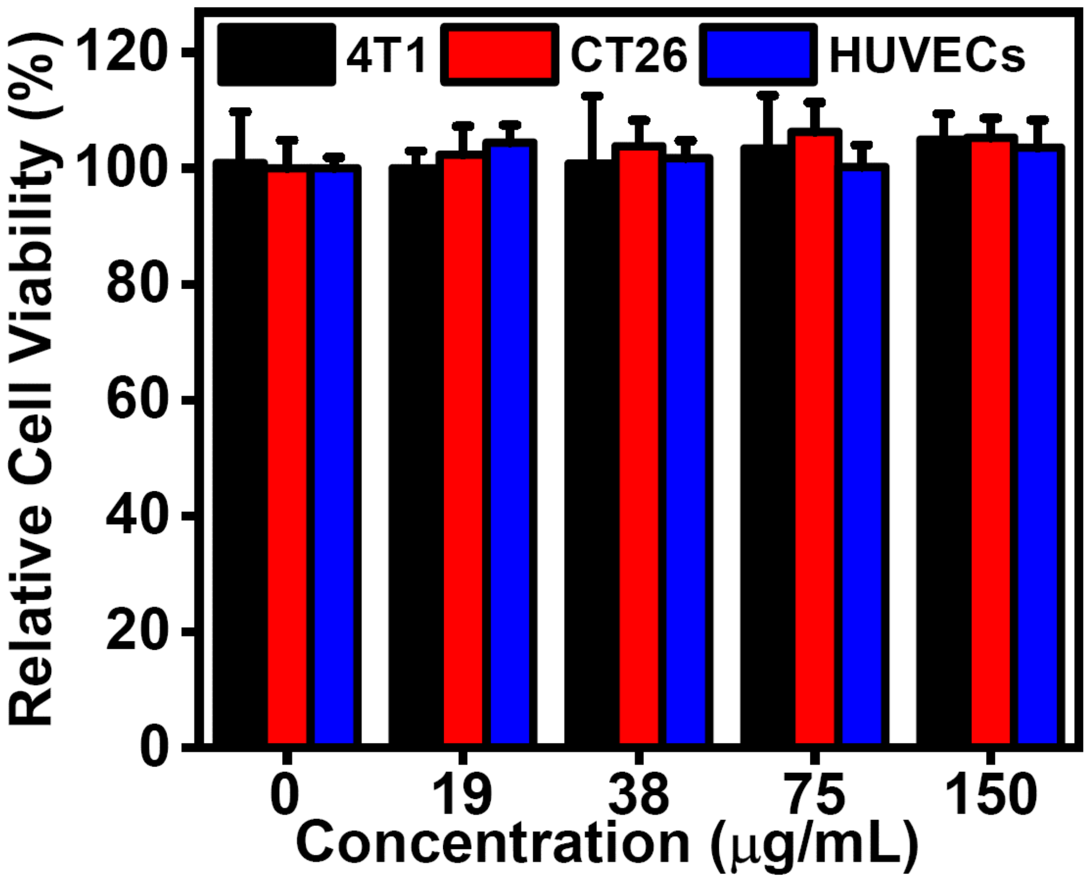

Figure S12. The relative cell viabilities after cultured with Ru-Phen CPNs for 12 hours.

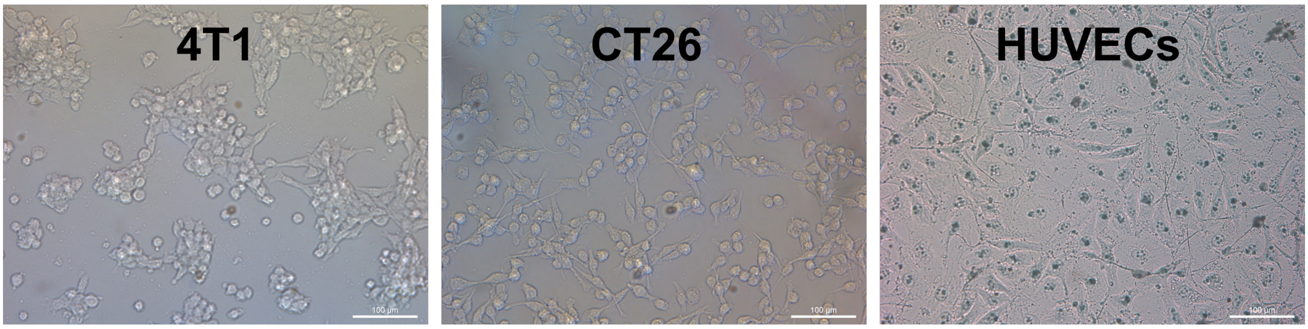

Figure S13. The optical photos of 4T1, CT 26, and HUVEC cells cultured with Ru-Phen CPNs for 12 hours.

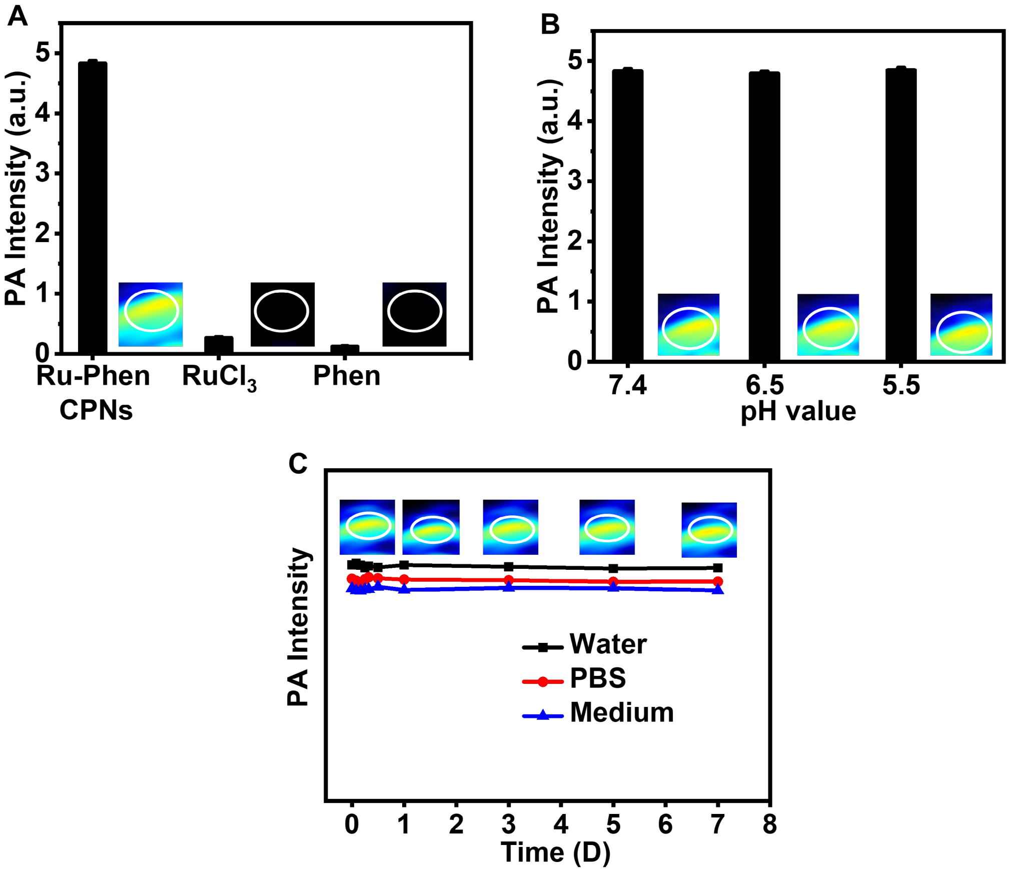

Figure S14. (**A**) The PA intensity of Ru-Phen CPNs, $RuCl_3$, and Phen. (**B**) PA intensity of Ru-Phen CPNs in different pH (7.4, 6.5, and 5.5). (**C**) The PA intensity of Ru-Phen CPNs in water, PBS, and medium (1640 cell medium + 10 % FBS) at different time points (0, 2 h, 4 h, 6 h, 8 h, 12 h, 1 D, 3 D, 5 D, and 7D).

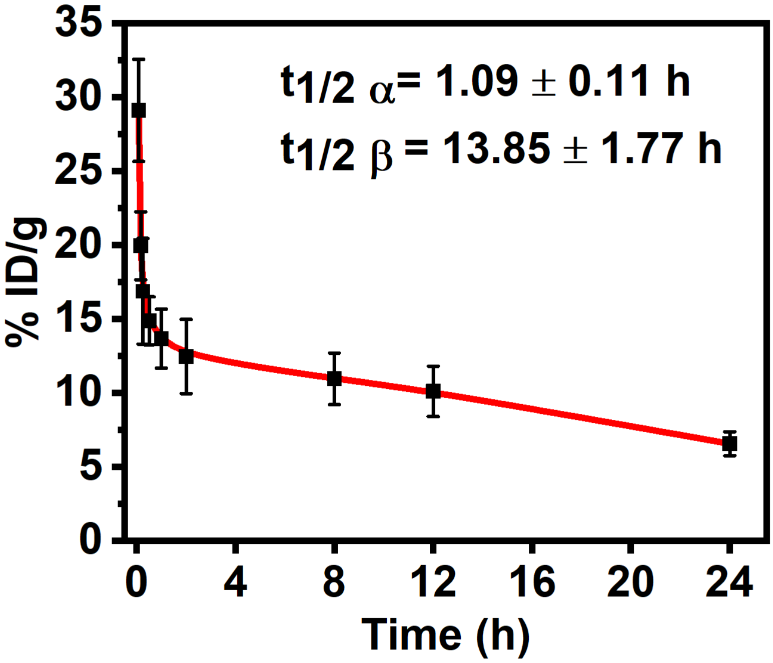

Figure S15. The blood circulation curve of Ru-Phen CPNs after intravenous injection.

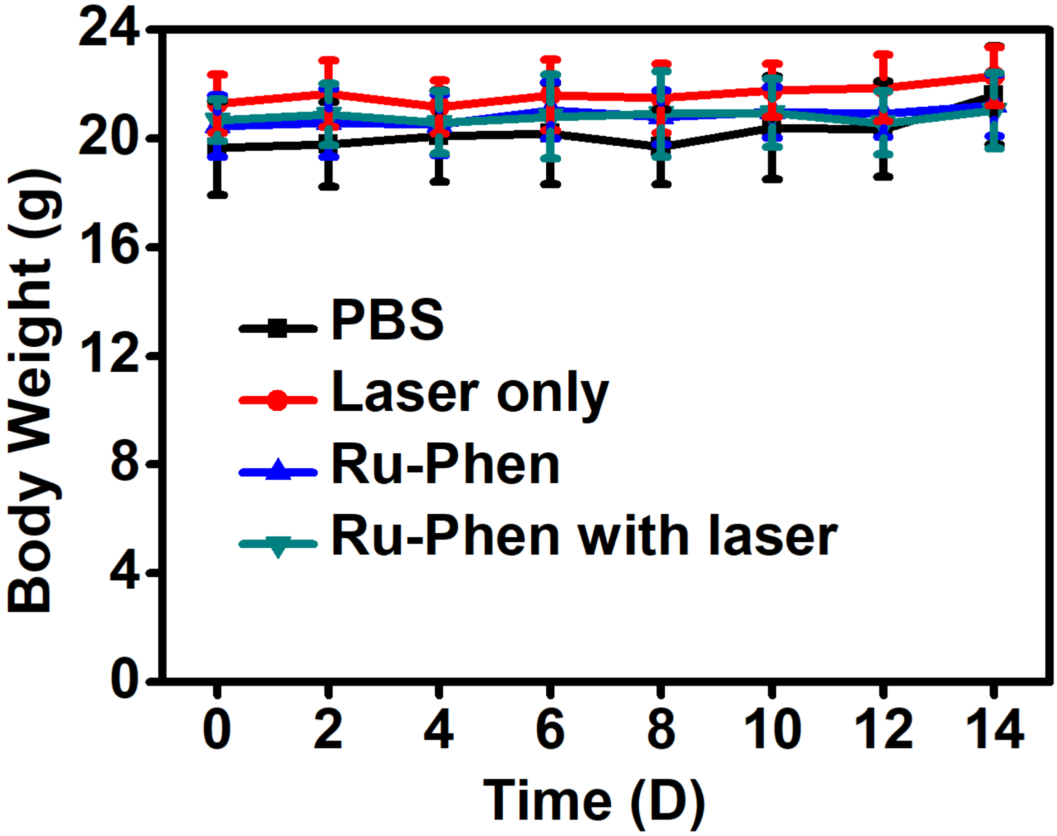

Figure S16 The body weights of mice with the various treatment.

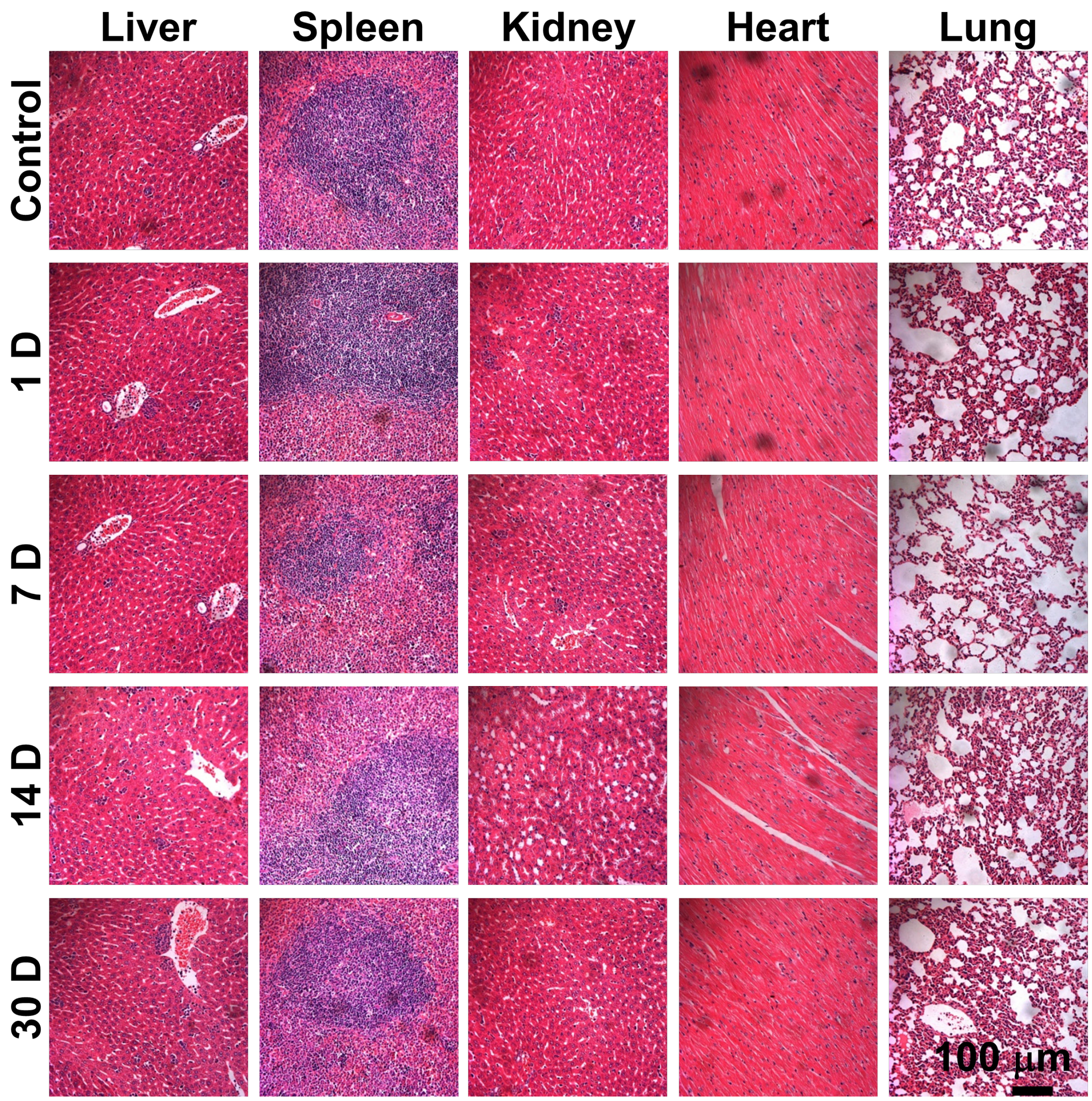

Figure S17. H&E staining of the major organs collected from the mice at 1, 7, 14, and 30 days post-injection of Ru-Phen CPNs .

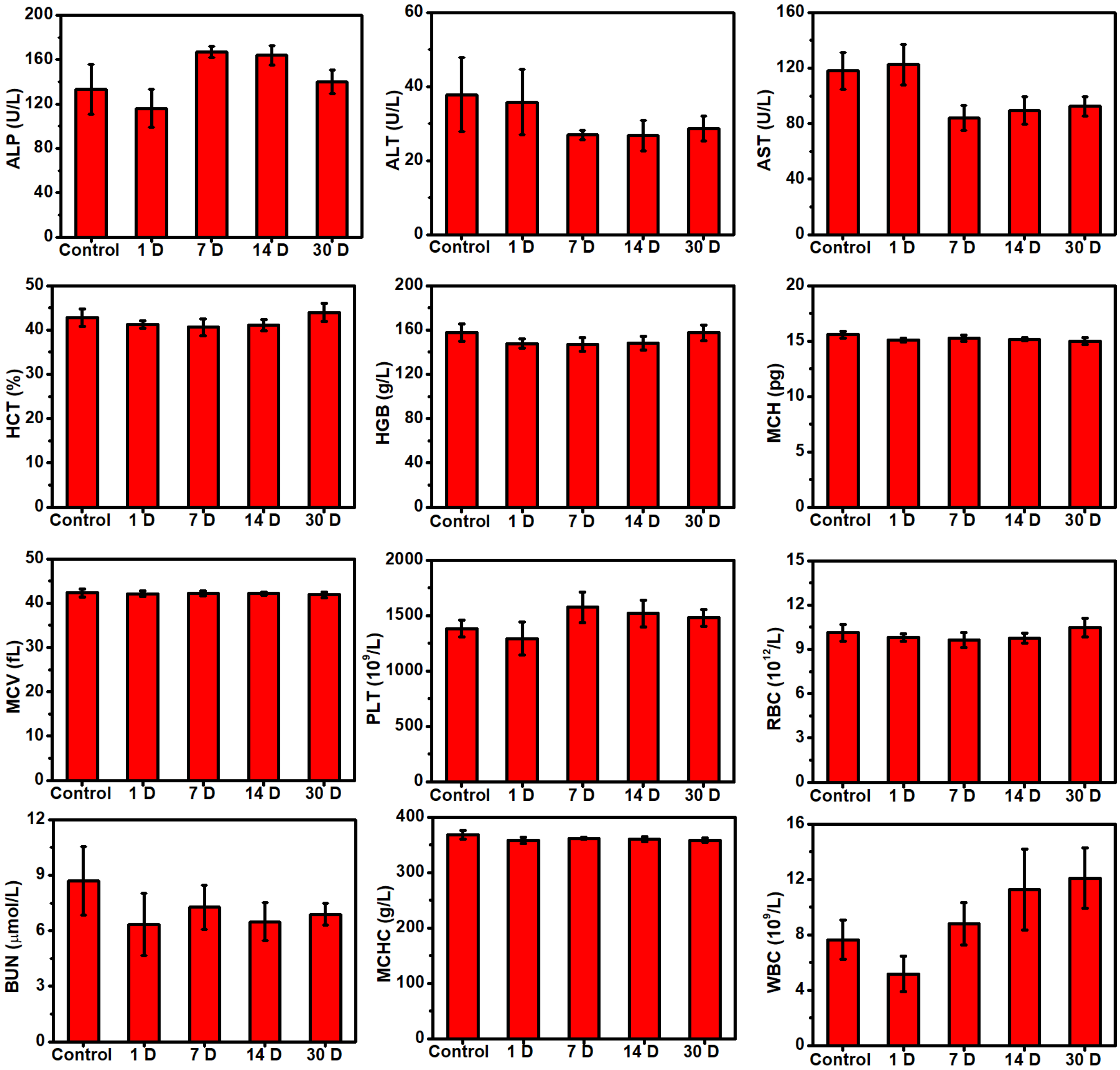

Figure S18. *In vivo* toxicology examination for mice treated with Ru-Phen CPNs. Healthy Balb/c mice intravenously injected with Ru-Phen CPNs were sacrificed at 1, 7, 14, and 30 days p.i. for blood collection. Serum biochemistry data including blood urea nitrogen (BUN) levels and liver function markers such as alkaline phosphatase (ALP), alanine aminotransferase (ALT), and aspartate aminotransferase (AST) were measured. Complete blood counts: hematocrit (HCT), hemoglobin (HGB), mean corpuscular hemoglobin (MCH), mean corpuscular hemoglobin concentration (MCHC), mean corpuscular volume (MCV), blood platelet (PLT), red blood cells (RBC), and blood levels of white blood cells (WBC) of control and Ru-Phen CPNs treated mice were also measured. The statistic was based on five mice per data point.
